# Novel Er^3+^/Nd^3+^-doped borofluoride glass materials for NIR optical fiber applications with suitable thermal and mechanical properties

**DOI:** 10.1038/s41598-026-67626-3

**Published:** 2026-09-15

**Authors:** Mona A. El Naggar, Aly Saeed, Heba A. Gohra

**Affiliations:** 1 Mathematics and Engineering Physics Department, Faculty of Engineering, Cairo University, Giza, Egypt; 2 Mathematical and Natural Science Department, Faculty of Engineering, Egyptian Russian University, Cairo, Egypt

**Keywords:** Optical fiber, photoluminescence, borofluoride glass, Er^3+^/ Nd^3+^, Chemistry, Materials science, Optics and photonics, Physics

## Abstract

To advance the performance of broadband optical fiber communication systems, a novel borofluoride-based glass system with the composition 60B_2_O_3_-20Pb_3_O_4_-10MgF_2_-10LiF (BPML) was synthesized and accordingly investigated. The base glass was doped with 1 mol% of Er^3+^, Nd^3+^, or their combination to produce BPML: RE^3+^ glass series. Glass structural analysis via X-ray diffraction (XRD), bulk density measurements, and Fourier transform infrared (FTIR) spectroscopy confirmed the amorphous nature of the glasses, indicating increased network compactness upon rare-earth doping, particularly with Er^3+^. The thermal properties were investigated through differential scanning calorimetry (DSC) and thermogravimetric analysis (TGA), revealing enhanced thermal stability across all synthesized doped glass samples. Furthermore, the theoretical estimation of the mechanical characterization, including predicted elastic moduli and hardness using the Makishima-Mackenzie model, showed rigidity improvements with the incorporation of RE^3+^. Optical absorption spectra recorded over the 400–1600 nm range reveal characteristic transitions of Er^3+^ and Nd^3+^, with co-doped samples displaying broader absorption bands, indicative of potential for wideband signal amplification. The photoluminescence studies under 773 nm excitation have demonstrated near-infrared (NIR) emission centered at 1.55 μm ($\:{f}_{0}=1.929\:x{10}^{14}$*Hz*). The co-doped synthesized samples have exhibited enhanced emission intensity, indicating efficient energy transfer. The present work results show that the synthesized samples, doped with Er^3+^, Nd^3+^, or their combination, exhibit an optimal balance of thermal stability, mechanical robustness, and broadband NIR emission. Evidently, the synthesized glass samples achieve a high-quality factor of ($\:{Q}_{f}=38.97$), underscoring their potential for high-performance broadband applications. These properties position the proposed samples as a promising material for the fabrication of NIR optical fibers for communication benefits.

**Supplementary Information:**

The online version contains supplementary material available at https://doi.org/10.1038/s41598-026-67626-3.

## Introduction

The rapid advancement of optical fiber technologies has significantly increased the demand for novel materials with tailored thermal, mechanical, and optical properties^1–2^. Near-infrared (NIR) emission in the (1.4–1.7) µm spectral region is of critical importance for advanced photonic technologies because of its favorable optical properties and compatibility with modern optoelectronic systems^1–2^. This spectral region includes the S, C, and L bands, which are foundational to fiber-optic communication, particularly around the 1.5 μm window. Ideal host materials for these applications should exhibit high mechanical rigidity, excellent thermal stability, besides the broad NIR emission characteristics^3–5^. Recently rare earth ions (RE^3+^), particularly erbium (Er^3+^) and neodymium (Nd^3+^), have emerged as crucial activators due to their well-defined transitions, long lifetime metastable states, and high quantum efficiency^6–8^. Specifically, the Er^3+^ ion is indispensable in modern optical communication systems, primarily due to its strong emission in the (1.5–1.6) µm range, corresponding to its ^4^I_13/2_→^4^I_15/2_ recorded transition^7–8^. Despite of the fact that Nd^3+^ is traditionally known for its intense emission at ~ 1.06 μm, its incorporation in Er^3+^/Nd^3+^ co-doped systems can significantly enhance the 1.55 μm emission through sensitization and energy-transfer processes. In such systems, the characteristic emission near 1.55 μm originates primarily from the ^4^I_13/2_→^4^I_15/2_ transition of Er^3+^, while Nd^3+^ ions act mainly as sensitizers that improve excitation efficiency and energy migration under suitable excitation conditions^4–6^. Borate-based glass samples represent a dependent versatile host matrix due to their low melting temperatures, high thermal and chemical stability, superior rare-earth ion solubility, and beside their high optical clarity^9–10^. The ability of B^3+^ ions to adopt both trigonal (BO_3_) and tetrahedral (BO_4_) coordination states facilitates the formation of a highly adaptable and compact network, which can be tailored structurally to accommodate dopant ions^11–12^. The incorporation of lead oxide (PbO) into borate glass significantly enhances its nonlinear optical properties by increasing the polarizability and promoting structural modifications via BO_3_↔BO_4_ interconversion. This transformation, coupled with the inherently high refractive index and optical clarity of lead borate glasses, renders them highly suitable for applications in lasers, optical switches, and nonlinear optical devices^13^. To overcome the high phonon energy typically associated with oxide glasses, which can quench rare-earth emission, halide ions such as fluoride ($\:{\mathrm{F}}^{-}$) are introduced to form oxyfluoride glasses^14–15^. Additives like MgF_2_ and LiF contribute to improve structural stability and the ionic conductivity while maintaining a low phonon energy environment, which is advantageous for efficient luminescence. Fluoride ions ($\:{\mathrm{F}}^{-}$) reduce the glass phonon energy by partially modifying the local oxygen coordination, while Mg^2+^ and Li^+^ promote network stabilization and structural rearrangement. These modifications create a more favorable local environment for Er^3+^ and Nd^3+^ ions by improving their dispersion and reduced non-radiative energy losses, thereby enhancing near-infrared luminescence and energy transfer efficiency^16–17^. Consequently, hybrid oxyfluoride glasses offer a balanced combination of thermal robustness, mechanical integrity, and enhanced photoluminescence performance^14,18,19^. Accordingly, rare earth-doped borate-based oxyfluoride glasses have attracted considerable attention as promising materials for advanced photonic and near-infrared optical applications.

With the increasing demand for advanced photonic devices, near-infrared (NIR) emitting glass materials have attracted considerable attention due to their applications in optical amplifiers, laser, and broadband communication systems. Previous studies have demonstrated that rare-earth-doped glasses, particularly Er^3+^ and Nd^3+^ containing systems, can provide efficient NIR emission through characteristic transitions. Elsaghier et al. (2022)^20^ reported Er^3+^-doped alkaline earth titanium borate glasses with broad emission around 1530–1565 nm, showing potential for optical amplifiers; however, the study was mainly limited to Er^3+^ emission without investigating the synergistic effect of multiple rare-earth ions. Farouk et al. (2013)^21^ investigated Er^3+^-containing bismuth borate glasses and focused primarily on structural modification and optical absorption behavior, while detailed NIR emission optimization was not explored. Nd^3+^-doped MgO-Al_2_O_3_-SiO_2_ transparent glass developed by Han et al. (2019)^22^ exhibited strong emissions at 898, 1057, and 1330 nm, making them promising for solid-state laser applications; however, their emission was mainly restricted to Nd^3+^ transitions and required a glass structure, which may complicate fabrication processes. Furthermore, Ding et al. (2023)^23^ achieved ultra-broadband NIR emission (1300–1630 nm) in Nd^3+^/ Tm^3+^ /Er^3+^ tri-doped tellurite glasses through energy transfer mechanisms, demonstrating excellent potential for broadband optical amplifiers. However, the requirements of multiple rare-earth dopants and relatively complex optimization of dopant concentrations may limit practical applications. Similarly, Er^3+^/Nd^3+^ reinforced lead-borate glasses and Nd^3+^-dopant gadolinium calcium silica borate glasses have shown strong NIR emission at 1059 nm and improved laser characteristics, but their emission regions are mainly concentrated at specific laser wavelengths rather than providing broad emission coverage in telecommunication window as reported by Kesavulu et al. (2016)^24^. Despite these significant advances, several challenges remain, including achieving broad and intense NIR emission around the low-loss optical communication region (1550 nm), improving emission bandwidth through efficient rare-earth energy transfer, and simultaneously maintaining good thermal, mechanical, and structural stability of the glass host. Many reported systems focus either on single rare-earth emission, laser-specific wavelengths, or require complex multi-component doping strategies, while fewer studies have explored a simple borate-based glass system capable of combining strong Er^3+^/Nd^3+^ emission, broadband characteristics and enhanced physical stability. Therefore, developing a stable borate-based Er^3+^/Nd^3+^ co-doped borate-based glass system with efficient energy transfer, broadened NIR emission, and high optical quality factor remains an important research direction for next-generation broadband photonic devices, optical amplifiers, NIR filters, and photonic communication applications.

This work concentrates on the design and synthesis of a novel borate-based oxyfluoride glass system with the composition, 60B_2_O_3_-20Pb_3_O_4_-10MgF_2_-10LiF, strategically doped with Er^3+^ and Nd^3+^ ions, both individually and in combination with varying concentrations (BPML: RE^3+^ glass series). The primary objective of this study to investigate the influence of Er^3+^, Nd^3+^, and their co-doping Er^3+^→Nd^3+^ on the structural, thermal, mechanical, and near-infrared optical properties of the developed glass system, with particular emphasis on broadband NIR emission and rare earth energy transfer mechanism. The novelty of this work lies in the development of a borate-rich hybrid oxyfluoride glass network that integrates the high rare earth ion solubility of borate glasses with the reduced phonon energy imparted by fluoride components, while simultaneously improving thermal and mechanical stability. The study further aims to establish structure-property relationships that clarify the role of rare earth incorporation in governing the physical and optical behavior of the glass network. The outcomes of this work are expected to provide valuable design guidelines for development of advanced rare earth doped borate-based oxyfluoride glasses for NIR photonic applications, while serving as a foundation for future investigations toward practical optical fiber implementation.

## Experimental procedures

### Glass synthesis

**Fig. 1:**
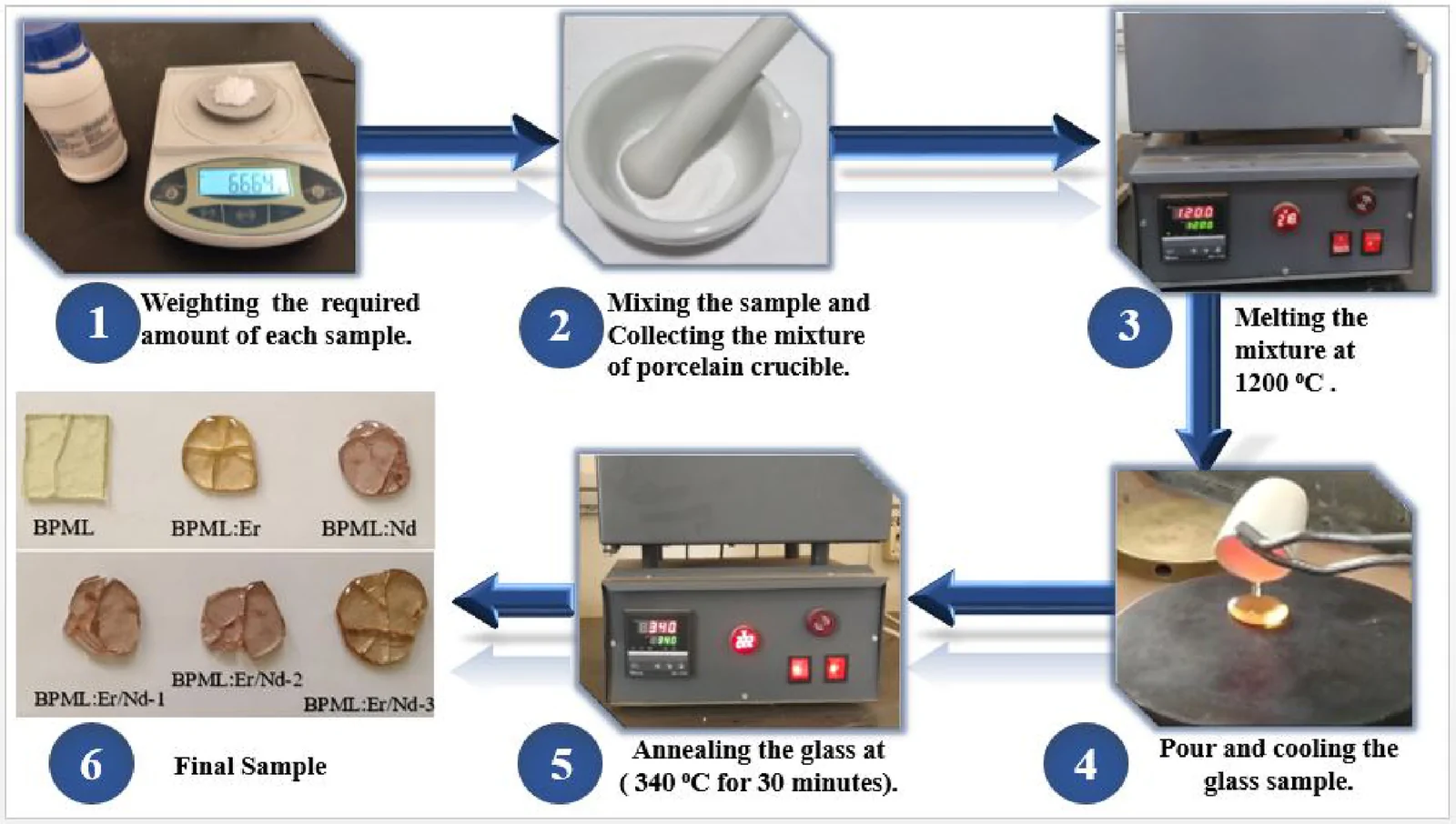
Schematic representation of the BPML and BPML: RE^3+^ preparation process (real-time photos).

The novel borate-based oxyfluoride glass system, 60B_2_O_3_-20Pb_3_O_4_-10MgF_2_-10LiF, was synthesized as a base sample. Afterwards, it was doped with RE^3+^, particularly Er^3+^, Nd^3+^ or both, as illustrated in Table 1. The system was synthesized using the melt-quenching technique. High-purity raw materials of H_3_BO_3_ (99.99%), Pb_3_O_4_ (99.99%), MgF_2_ (99.98%), LiF (99.98%), Er_2_O_3_ (99.99%), and Nd_2_O_3_ (99.99%) were procured from Sigma-Aldrich. The precursors were weighed precisely according to the target molar ratios to prepare a total batch weight of 25 g for each glass composition and thoroughly mixed in a porcelain mortar for one hour to ensure compositional uniformity. To compensate for the possible volatilization of fluoride species during melting, the weighed amounts of LiF and MgF_2_ were each increased by 10 wt% relative to their calculated baches weights. This processing excess was introduced to minimize fluoride loss and maintain the final glass composition as close as possible to the nominal composition. The batches were introduced into an alumina crucible and placed directly in furnace maintained at 1200 °C, where they are melted for one hour under ambient air atmosphere. The 1200 °C was selected based on previous studies on lead borate/ oxyfluoride glasses containing rare earth and preliminary melting trials, which confirmed complete melting, good melt homogeneity, and the absence of visible undissolved particles. During the melting, the molten glass was manually stirred several times to improve compositional homogeneity and facilitate complete dissolution of the raw materials. As Pb_3_O_4_ is thermally unstable above ~ 500 °C, it decomposes to PbO and O_2_ during melting. The resulting PbO participates directly in glass formation, contributing to network modification and increasing polarizability. Therefore, Pb_3_O_4_ effectively functions as a PbO precursor under the provided synthesis conditions. The compositions listed in Table 1 correspond to the nominal batch compositions. Pb_3_O_4_ was used as the starting lead precursor, and its decomposition into PbO with oxygen evolution is an intrinsic part of the melting process. Since the study is based on the nominal batch compositions, no additional correction associated with oxygen evolution was introduced during the batch calculations. The viscous molten glass was rapidly cast into a preheated stainless-steel mold and immediately transferred to a furnace maintained at 340 °C for 30 min to relieve thermal stresses. The annealing temperature and duration were optimized experimentally using polarized light microscopy to monitor residual thermal stresses. Several annealing conditions with different temperatures and holding times were evaluated. Initial treatments at lower temperatures did not adequately relieve the internal stresses, even when the annealing time was extended. Consequently, the annealing temperature was progressively increased, and the stress state was examined after each trial. Based on these observations, an annealing treatment at 340 °C for 30 min was selected as the optimum condition, ensuring efficient stress relaxation while preserving the amorphous nature of the glass. Figure 1 illustrates the synthesis process schematically, as shown in real-time photos. All prepared glass samples were visually inspected to evaluate their macroscopic quality prior to characterization. As shown in Fig. 1, the obtained glasses were transparent and exhibited good visual homogeneity without observable nature cracks, bubbles, or macroscopic evidence of phase separation.

**Table 1:** The composition of the synthesized BPML and BPML: RE^3+^ glass samples.

Glass sample code	Elemental composition					
	B_2_O_3_	Pb_3_O_4_	MgF_2_	LiF	Er_2_O_3_	Nd_2_O_3_
BPML	60	20	10	10	0	0
BPML: Er	60	20	10	10	1	0
BPML: Nd	60	20	10	10	0	1
BPML: Er/Nd-1	60	20	10	10	0.5	0.5
BPML: Er/Nd-2	60	20	10	10	0.25	0.75
BPML: Er/Nd-3	60	20	10	10	0.75	0.25

### Structural examinations

X-ray diffraction (XRD), density measurements, density-related parameters, and Fourier transform infrared (FTIR) spectra were all performed and analyzed to study the structural modifications resulting from Er^3+^ and Nd^3+^ doping process. XRD patterns were recorded by a Philips X-ray diffractometer with a wavelength of 1.54056 Å from a Cu-Kα filter. For XRD analysis, representative glass samples were crushed and finely ground into homogeneous powders using an agate mortar to eliminate any influence of sample geometry and ensure reliable diffraction measurements. The bulk density ($\:{\uprho\:}$) of the fabricated BPML: RE^3+^ glass samples was rigorously assessed according to the ASTM C373 standard. Density measurements were carried out on randomly selected bulk glass pieces without imposing a specific geometry or dimensions, since the Archimedes method depends only on the sample mass in air and the immersion liquid. Key structural parameters, including molar volume ($\:{\mathrm{V}}_{\mathrm{m}}$), mean boron-boron separation ($\:{\mathrm{d}}_{\mathrm{B}-\mathrm{B}}$), oxygen packing density (OPD), and packing density ($\:{\mathrm{V}}_{\mathrm{t}}$), were calculated via Eqs. 1–4 to evaluate the effects of Er^3+^, Nd^3+^, and co-doping (Er^3+^/Nd^3+^) on the glass network^25–26^.

1 $$\:{\mathrm{V}}_{\mathrm{m}}=\frac{\mathrm{M}}{{\uprho\:}}\:$$

where, $\:\mathrm{M}$ is molecular mass of the glass sample.

2 $$\:{\mathrm{d}}_{\mathrm{B}-\mathrm{B}}={\left(\frac{{\mathrm{V}}_{\mathrm{m}}^{\mathrm{B}}}{{\mathrm{N}}_{\mathrm{A}}}\right)}^{1/3}\:$$

where, $\:\:{\mathrm{V}}_{\mathrm{m}}^{\mathrm{B}}$is the volume of one mol of B^3+^ ions within the considered glass sample, and $\:{\mathrm{N}}_{\mathrm{A}}\:$is the Avogadro’s number.

3 $$\:\mathrm{O}\mathrm{P}\mathrm{D}=\mathrm{n}\frac{{\uprho\:}}{\mathrm{M}}$$

where, $\:\mathrm{n}\:$refers to the number of oxygen atoms per chemical formula.

4 $$\:{\mathrm{V}}_{\mathrm{t}}=\frac{{\uprho\:}}{\mathrm{M}}\sum\:_{\mathrm{i}}{\mathrm{x}}_{\mathrm{i}}{\mathrm{V}}_{\mathrm{i}}$$

where, $\:{\mathrm{x}}_{\mathrm{i}}$ is the mole fraction of the sample constituents, and$\:\:{\mathrm{V}}_{\mathrm{i}}$ is the packing factor.

The values of $\:{\mathrm{V}}_{\mathrm{m}}^{\mathrm{B}}$ and $\:{\mathrm{V}}_{\mathrm{i}}$ were calculated using the following relations.

5 $$\:{\mathrm{V}}_{\mathrm{m}}^{\mathrm{B}}=\frac{{\mathrm{V}}_{\mathrm{m}}}{2\left(1-{\mathrm{X}}_{\mathrm{B}}\right)}\:$$

6 $$\:\:\:\:\:\:\:\:\:\:{\mathrm{V}}_{\mathrm{i}}=\frac{4{\uppi\:}{\mathrm{N}}_{\mathrm{A}}}{3}\left(\mathrm{b}{\mathrm{r}}_{\mathrm{A}}^{3}+\mathrm{c}{\mathrm{r}}_{\mathrm{O}}^{3}\right)$$

where, $\:{\mathrm{X}}_{\mathrm{B}}$ is the molar fraction of $\:{\mathrm{B}}_{2}{\mathrm{O}}_{3}$,$\:\:{\mathrm{r}}_{\mathrm{A}}$ is the ionic radius of the cation and $\:{\mathrm{r}}_{\mathrm{O}}$ the ionic radius of the anion of the oxide element $\:{\mathrm{A}}_{\mathrm{b}}{\mathrm{O}}_{\mathrm{c}}$.

It should be noted that the packing density calculations were carried out using Shannon ionic radii based on the assumption of fixed ionic coordination and spherical ions. Nevertheless, in borate-based glass systems, the effective ionic radii, particularly for B^3+^ species, are strongly influenced by the local coordination environment associated with BO_3_ and BO_4_ structural units. Consequently, the calculated packing-related parameters should be interpreted as representative indicators of the relative structural compactness and network evolution among the investigated glass compositions.

Furthermore, the FTIR spectra were recorded in the 400–4000 cm^− 1^ range, with spectral resolution ± 2, using a JASCO FTIR6200. For FTIR measurements, the glass samples were finely ground into powders to ensure homogeneous mixing with KBr. A mass of 3.00 mg for all synthesized samples was homogeneously mixed with 300 mg of dry KBr and compressed under a compression load of 13 tons to produce transparent pellets suitable for analysis. The broad absorption bands in the FTIR spectra were deconvoluted using the Gaussian fitting. The minimum number of component bands required to reproduce the experimental spectra was employed while preserving physically meaningful band positions consistent with the characteristic vibrational modes reported for multicomponent borate glasses. During the fitting procedure, peak positions, widths, and intensities were allowed to vary within physically reasonable limits, and no artificial peaks were introduced solely to improve the mathematical fit. The quality of the fitting was evaluated using the coefficient of determination ($\:{R}^{2}$), reduced chi-squared ($\:{\chi\:}^{2}$), and inspection of the residuals between the experimental and fitted spectra. The proportions of tetrahedral BO_4_ (N_4_) and trigonal BO_3_ (N_3_) structural units were calculated according to Eqs. 7 and 8^27–28^.

7 $$\:{N}_{4}=\frac{{A}_{2}}{{A}_{1}+{A}_{2}}$$

8 $$\:{N}_{3}=\frac{{A}_{1}}{{A}_{1}+{A}_{2}}$$

where, A_1_$\:\:\equiv\:\:$area (BO_4_) and A_2_$\:\:\equiv\:\:$area (BO_3_) denotes the integrated areas of the deconvoluted BO_4_ and BO_3_ peaks, respectively. It should be note that the calculated $\:{N}_{4}$ and $\:{N}_{3}$ values are considered semi-quantitative because the absorption coefficients and oscillator strengths of BO_3_ and BO_4_ structural units are not identical. Therefore, direct comparison of the integrated FTIR band areas may introduce systematic deviations in the absolute values of the calculated fractions. Nevertheless, the adopted approach remains useful for evaluating the relative structural variations and comparative trends among the investigated glass compositions.

The force constant, which quantifies the bond rigidity was calculated using Eq. 9 to accurately examine the observed vibrational shifts^29–30^.

9 $$\:\nu\:=\frac{1}{2c\pi\:}\sqrt{\frac{k}{\mu\:}}\:$$

Here, $\:\nu\:$ stands for vibration frequency, * c* represents speed of light, and $\:\mu\:$ denotes reduced mass (kg). the calculation of $\:\mu\:$ was performed using the relationship outlined in Eq. 10.

10 $$\:\mu\:=\frac{{m}_{o}{m}_{B}}{{m}_{0}+{m}_{B}}$$

Here, $\:{m}_{o}$ is the mass of oxygen and $\:{m}_{B}$ is the mass of boron atom bounded to the oxygen atom.

### Thermal properties examination

The thermal behavior of the synthesized BPML: RE^3+^ glass samples was assessed using the differential scanning calorimetry (DSC) and the thermogravimetric analysis (TGA). For thermal characterization, representative glass samples were crushed into fine powders prior to DSC and TGA measurements to ensure good thermal contact and unform heat transfer throughout the analysis. The DSC was mainly employed to determine critical thermal transition parameters, including the glass transition temperature ($\:{\mathrm{T}}_{\mathrm{g}}$), onset of crystallization ($\:{\mathrm{T}}_{\mathrm{c}}$), peak crystallization temperature ($\:{\mathrm{T}}_{\mathrm{p}}$), and melting temperature ($\:{\mathrm{T}}_{\mathrm{m}}$). These parameters provide essential insights into the thermal stability, phase transformation behavior, and glass processing. Complementarily, TGA was utilized to evaluate the glass samples’ thermal stability and compositional integrity. DSC and TGA measurements were performed simultaneously using an SDT Q600 thermal analyzer (TA Instruments, USA) under a flowing nitrogen atmosphere (15 psi), ensuring an inert environment to prevent oxidation. The heating rate was maintained at 10 °C/min from ambient temperature to 1000 °C.

### Elastic properties calculation

The theoretical elastic properties, Young’s modulus ($\:\mathrm{E}$), bulk modulus ($\:\mathrm{K}$), shear modulus ($\:\mathrm{G}$), and longitudinal modulus ($\:\mathrm{L}$), in addition to Poisson’s ratio ($\:{\upsigma\:}$) and hardness ($\:\mathrm{H}$) were evaluated using the semi-empirical Makishima-Mackenzie model, which relates macroscopic elastic behavior to dissociation energies and packing densities of the constituent oxide units. Within this framework, the elastic response of the glass network, when expressed in terms of Young’s modulus ($\:\mathrm{E}$), is given by

9 $$\:\mathrm{E}=2{\mathrm{V}}_{\mathrm{t}}{\mathrm{G}}_{\mathrm{t}}$$

where, $\:{\mathrm{G}}_{\mathrm{t}}$ is the total dissociation energy per unit volume of the glass sample and thus calculated by:

10 $$\:{\mathrm{G}}_{\mathrm{t}}=\sum\:_{\mathrm{i}}^{\mathrm{n}}{\mathrm{G}}_{\mathrm{i}}{\mathrm{x}}_{\mathrm{i}}$$

where, * n* is the number of constituent oxides, $\:{\mathrm{G}}_{\mathrm{i}}$ is the bond energy per volume of the $\:{i}^{th}$ oxide, and $\:{\mathrm{x}}_{\mathrm{i}}$ is its molar fraction.

Hence, the bulk modulus (* K*), shear modulus (G), longitudinal modulus (L), Poisson’s ratio ($\:\sigma\:$), and hardness (H) are determined using the following relations^31–32^:

11 $$\:\mathrm{K}=1.2{\mathrm{V}}_{\mathrm{t}}\mathrm{E}$$

12 $$\:\mathrm{G}=\frac{3\mathrm{E}\mathrm{K}}{9\mathrm{K}-\mathrm{E}}$$

13 $$\:\mathrm{L}=\mathrm{K}+\left(\frac{4\mathrm{G}}{3}\right)$$

14 $$\:{\upsigma\:}=\frac{\mathrm{L}-2\mathrm{G}}{2(\mathrm{L}-\mathrm{G})}$$

15 $$\:\mathrm{H}=\frac{\left(1-2{\upsigma\:}\right)\mathrm{E}}{6\left(1+{\upsigma\:}\right)}$$

### Optical properties examination

For optical absorption and photoluminescence measurements, bulk glass specimens were polished on both faces using successive SiC abrasive papers until smooth, scratch-free optical surfaces were obtained. The polishing process was continued until the sample thickness reach approximately 1.2–1.3 mm. Since the optical measurements depend primarily on the optical path length, the lateral dimensions of the specimens were not fixed and were selected according to the available glass pieces. The optical characteristics of the synthesized BPML: RE^3+^ glass samples were examined across the visible-NIR spectral region. Absorption spectra were recorded using a JASCO V-670 UV-Vis-NIR spectrophotometer, operating with a spectra resolution of 2 nm, ensuring high measurement accuracy and reproducibility.

The quantitative analysis of the crystal-field transitions and local environment asymmetry was evaluated via the Judd-Ofelt (J-O) theory. The experimental oscillator strengths ($\:{f}_{exp}$) for the identified $\:{Er}^{3+}$absorption bands were determined from the integrated absorption coefficients using the relation^11,15^:

16 $$\:{f}_{exp}=\frac{4{m}_{e}{c}^{2}{\epsilon}_{0}}{{e}^{2}N}\:\int\:\alpha\:\left(v\right)dv$$

where $\:{m}_{e}$ and * e* are the electron mass and charge, * c* is the velocity of light, $\:{ϵ}_{0}$ is the vacuum permittivity, * N* is the $\:{Er}^{3+}$ ion concentration ($\:\mathrm{i}\mathrm{o}\mathrm{n}\mathrm{s}/{cm}^{3}$), and $\:\alpha\:\left(v\right)$ is the absorption coefficient at frequency * v*. According to the J-O model, the theoretical oscillator strengths ($\:{f}_{calc}$) for electric-dipole transitions from the ground state ($\:\psi\:J$) to an excited state ($\:{\psi\:}^{{\prime\:}}{J}^{{\prime\:}}$) are expressed as^11,15^:

17 $$\:{f}_{calc}=\frac{8{\pi\:}^{2}{m}_{e}cv}{3h(2J+1)}\:\left[\frac{{({n}^{2}+2)}^{2}}{9n}\right]\sum\:_{k=\mathrm{2,4},6}{{\Omega\:}}_{k}{\left| \langle \psi\:J\left|\right|{U}^{\left(k\right)}\mid {\psi\:}^{{\prime\:}}{J}^{{\prime\:}} \rangle\right|}^{2}$$

where * h* is the Plank’s constant, * n* is the refractive index of the glass matrix, $\:\left[{\left({n}^{2}+2\right)}^{2}/9n\right]$ represents the local field correction factor, and $\:{\left|\langle\psi\:J\left|\right|{U}^{\left(k\right)}|{\psi\:}^{{\prime\:}}{J}^{{\prime\:}} \rangle\right|}^{2}$ are the reduced matrix elements for $\:{Er}^{3+}$ transitions. The phenomenological J-O intensity parameters ($\:{{\Omega\:}}_{2},\:{{\Omega\:}}_{4},\:{{\Omega\:}}_{6}$) were extracted by minimizing the root-mean-square (RMS) deviation between $\:{f}_{exp}$ and $\:{f}_{calc}$ using a standard least-squares fitting procedure.

### Photoluminescence

Photoluminescence measurements of the synthesized BPML: RE^3+^ glass samples were performed using an Edinburgh Instruments FLS1000 fluorescence spectrometer. Steady-state spectra were recorded using a continuous-wave xenon arc lamp as the excitation source, where the excitation wavelength was set at 773 nm using a monochromator.

The quantitative parameters governing the $\:{Nd}^{3+}\to\:{Er}^{3+}$ energy transfer were evaluated using the Förster-Dexter model. The spectral overlap integral (J) was calculated via^33–34^:

18 $$\:J=\int\:{f}_{D}\left(\lambda\:\right){\epsilon\:}_{A}\left(\lambda\:\right){\lambda\:}^{4}d\lambda\:\:$$

where $\:{f}_{D}\left(\lambda\:\right)$ is the normalized donor emission spectrum and $\:{\epsilon\:}_{A}\left(\lambda\:\right)$is the acceptor molar absorption coefficient. The critical transfer distance ($\:{R}_{0}$) was subsequently estimated using the relation^33–34^:

19 $$\:{R}_{0}^{6}=\frac{3{h}^{4}{c}^{4}{\eta\:}_{Q}}{4{\pi\:n}^{4}}J$$

where * h* is the Plank’s constant, * c* is the velocity of light, * n* is the refractive index, and $\:{\eta\:}_{Q}$ is the donor quantum efficiency.

## Results and discussion

### Structural properties

#### X-ray diffraction spectra

**Fig. 2:**
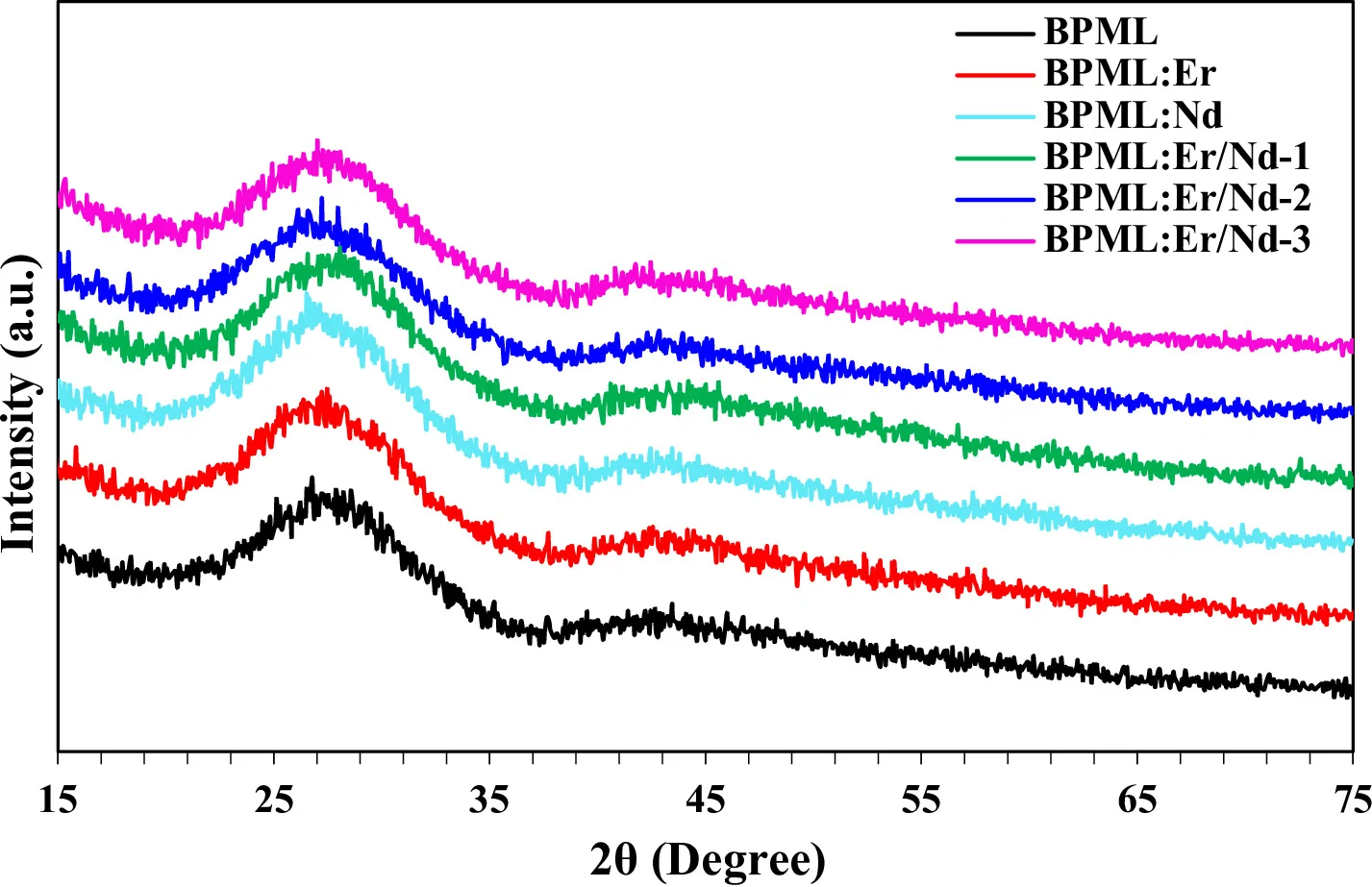
The recorded XRD spectra of the BPML and BPML: RE^3+^ glass series.

The broad, diffuse halos observed in the XRD spectra of the synthesized BPML: RE^3+^ glass series, as shown in Fig. 2, are features of an amorphous structure, confirming the lack of long-term crystalline order. Notably, no appreciable changes in the diffraction patterns were detected with varying concentrations of Er_2_O_3_ and Nd_2_O_3_, indicating stability of the amorphous structure.

#### Bulk density measurements

The structural parameters of the BPML: RE^3+^ glass series, characterized by bulk density ($\:{\uprho\:}$), molar volume ($\:{\mathrm{V}}_{\mathrm{m}}$), average boron-boron separation ($\:{\mathrm{d}}_{\mathrm{B}-\mathrm{B}}$), oxygen packing density ($\:\mathrm{O}\mathrm{P}\mathrm{D}$), and packing density ($\:{\mathrm{V}}_{\mathrm{t}}$) are listed in Table 2. The results indicate that doping the base sample (BPML) with Er^3+^, Nd^3+^, or their combination leads to a noticeable increase in density compared to the undoped sample. This behavior reflects the combined influence of the relatively high atomic masses of the incorporated rare earth ions and the structural evaluation of the glass network induced by rare earth incorporation. Among all compositions, BPML: Er glass exhibits the highest density, which is consistent with the larger atomic mass of Er^3+^ together with smaller ionic radius and strong field strength, promoting a more efficient atomic arrangement within the glass matrix. The stronger field strength of Er^3+^ originates from its smaller ionic radius relative to Nd^3+^. According to Shannon’s effective ionic radii for six-fold coordination, the ionic radii of Er^3+^ and Nd^3+^ are approximately 0.89 and 0.98 Å, respectively. Consequently, Er^3+^ possesses a higher charge-to-radius ratio (field strength), resulting in stronger electrostatic interactions with surrounding oxygen ions, enhanced $\:\mathrm{R}\mathrm{E}-\mathrm{O}$ bond strength, and more efficient atomic packing within the borate glass network^35–38^. These characteristics promote an efficient atomic arrangement and tighter network connectivity, resulting in increased structural compactness relative to both the Nd^3+^ doped and base samples. The corresponding decrease in molar volume $\:\left({V}_{m}\right)$, together with the decrease in the average boron-boron separation ($\:{d}_{B-B}$) and the increase in both oxygen packing density (* OPD*) and packing density ($\:{V}_{t}$), collectively indicates a progressive enhancement of the network packing efficiency following the Er^3+^ and Nd^3+^ incorporation. The BPML: Er exhibits the lowest molar volume, as observed in Table 2, reinforcing the role of Er^3+^ doping in enhancing network compactness. This behavior can be structurally rationalized by the high field strength of rare earth cations, which act as efficient topological constraints. The relatively small Er^3+^ ions occupy the interstitial voids within the borate network, acting as crosslinking centers that contract the free volume and maximize the packing efficiency of the glass matrix. Similarly, the slight decrease in the average boron-boron separation ($\:{\mathrm{d}}_{\mathrm{B}-\mathrm{B}}$) for doped glass samples, further confirms the formation of a denser local network. This reduction arises from the increased conversion of trigonal BO_3_ units into tetrahedral BO_4_ units^39–40^, as evidenced by the higher N_4_ (BO_4_ fraction) and lower N_3_ (BO_3_ fraction) values listed in Table 2. The incorporation of highly polarizing RE^3+^ ions induces a local charge compensation mechanism, forcing the borate matrix to shift its equilibrium toward the more stable and tightly bound BO_4_ configuration. The formation of BO_4_ tetrahedra introduces additional bridging oxygens, strengthening the network connectivity and reducing free volume. The N_4_ parameter represents the fraction of four-coordinated boron atoms (BO_4_), which contribute to a more polymerized and rigid glass network. In contrast, N_3_ corresponds to three-coordinated boron atoms (BO_3_) and less cross-linked structures. Therefore, an increase in N_4_ at the expense of N_3_ signifies enhanced structural polymerization and compactness. These values were derived from the deconvoluted FTIR spectra, discussed in detail below. Between the two dopants, Er^3+^ exerts a slightly stronger influence on structural densification, as indicated by its lower $\:{\mathrm{d}}_{\mathrm{B}-\mathrm{B}}$ value and higher OPD. This effect is attributed to Er^3+^’s higher polarizing power, which allows it to distort and compact the glass network more effectively than Nd^3+^. The progressive increase in both $\:\mathrm{O}\mathrm{P}\mathrm{D}$ and $\:{\mathrm{V}}_{\mathrm{t}}$ further corroborates the enhanced packing efficiency of the borate network following Er^3+^ and Nd^3+^ incorporation, in excellent agreement with the reduced molar volume, shorter average $\:\mathrm{B}-\mathrm{B}$ separation, and the increased BO_4_ fraction derived from FTIR analysis.

**Table 2:** The structural parameters of the synthesized glass samples: BPML and BPML: RE^3+^.

Glass sample code	$\:\boldsymbol{\uprho\:}$ (g/cm^3^)	$\:{\mathbf{V}}_{\mathbf{m}}$ (cm^3^/mol)	$\:{\mathbf{d}}_{\mathbf{B}-\mathbf{B}}$ (Å)	OPD	PD	$\:{\mathbf{N}}_{4}$	$\:{\mathbf{N}}_{3}$
BPML	3.418 ± 0.002	54.920	4.849	47.342	0.560	0.485	0.515
BPML: Er	3.497 ± 0.007	54.773	4.844	48.016	0.567	0.489	0.511
BPML: Nd	3.484 ± 0.009	54.845	4.847	47.953	0.566	0.532	0.468
BPML: Er/Nd-1	3.491 ± 0.004	54.801	4.845	47.992	0.566	0.532	0.468
BPML: Er/Nd-2	3.488 ± 0.004	54.815	4.846	47.979	0.566	0.512	0.483
BPML: Er/Nd-3	3.494 ± 0.001	54.787	4.845	48.004	0.567	0.543	0.457

#### FTIR spectroscopy

**Fig. 3:**
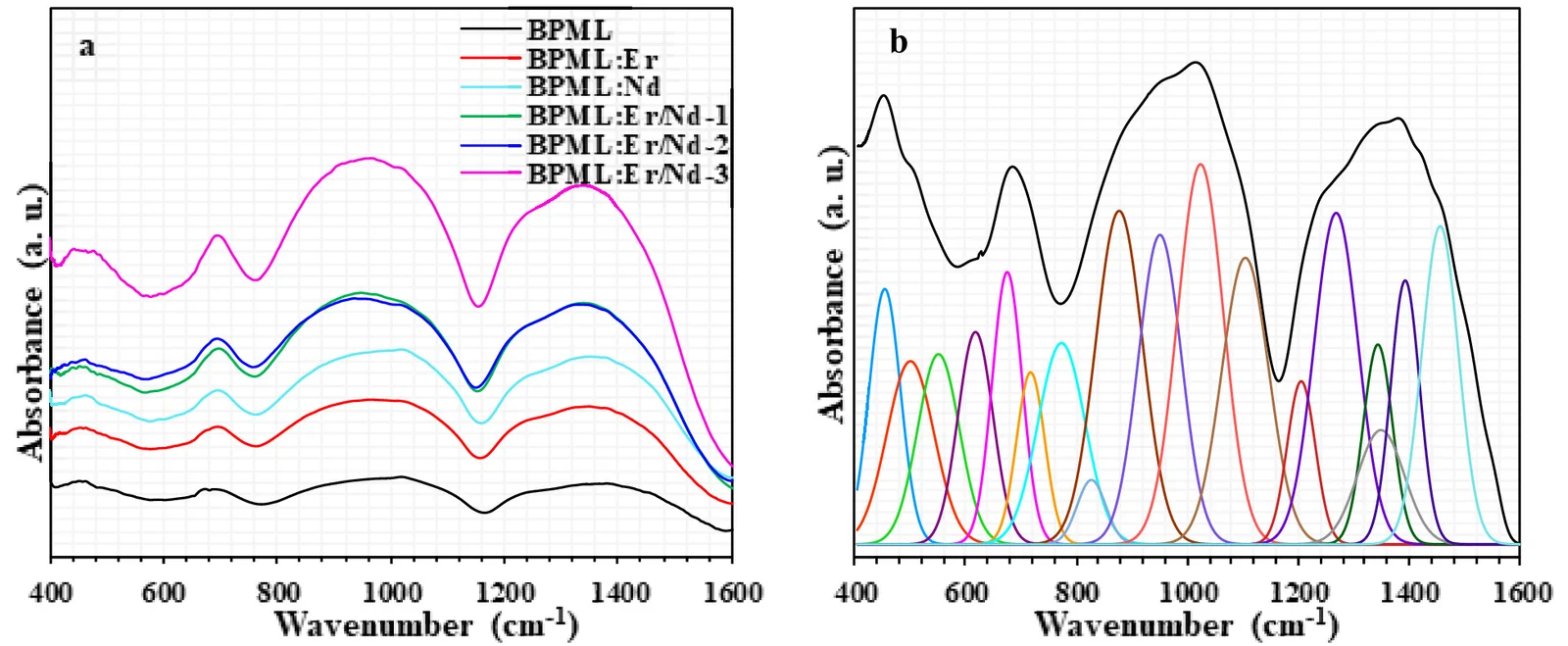
**a**) The FTIR spectra of the BPML and BPML: RE^3+^ glass series and **b**) the deconvolution of BPML glass as an example.

The FTIR is performed for all the synthesized samples and the produced spectra are shown in Fig. 3a versus wavelength. The FTIR spectra of the synthesized BPML: RE^3+^ glass series reveal the characteristic vibrational features of borate-based networks. Three main absorption regions can be identified, where:

- Wavelength 650–780 cm^-1^, is associated with the bending vibrations of BO_3_ units and $\:\mathrm{B}-\mathrm{O}-\mathrm{B}$ linkages.
- Wavelength 790–1150 cm^-1^, corresponds to $\:\mathrm{B}-\mathrm{O}$ stretching in BO_4_ tetrahedra and mixed BO_3_/BO_4_ structural units.
- Wavelength 1160–1590 cm^-1^, is attributed to asymmetric stretching modes of BO_3_ units, especially those containing non-bridging oxygens (NBOs).

Additionally, a distinct band in the (400–600) cm^-1^ region arises from vibrations involving metal-oxygen bonds ($\:\mathrm{P}\mathrm{b}-\mathrm{O}$, $\:\mathrm{L}\mathrm{i}-\mathrm{O}$, and $\:\mathrm{M}\mathrm{g}-\mathrm{O}$) within the glass matrix.

To gain deeper insight into the glass structure, Gaussian deconvolution was used to resolve the broad absorption bands and to identify the overlapping vibrational contributions associated with the different structural units present in the investigated glasses. Figure 3b presents the deconvoluted spectrum for the (BPML) sample as a representative example. The deconvolution process has resolved the broad envelope into multiple overlapping bands centered at 455, 501, 552, 618, 675, 718, 773, 827, 877, 951, 1024, 1104, 1205, 1268, 1343, 1392, and 1455 cm^− 1^. Table 3 illustrates the assignments of the multiple overlapping bands. Although the deconvolution resolved the broad FTIR envelope into multiple overlapping sub-bands, these bands should not be interpreted as independent structural entities. Rather, they represent overlapping vibrational contributions from the various borate structural units commonly reported in multicomponent borate glasses. The Gaussian fitting was performed using literature-based band assignments as initial constraints, while allowing only physically reasonable variations in peak position, width, and intensity to achieve the best fit. The excellent agreement between the experimental and fitted spectra, together with the very low residual errors and high goodness-of-fit values, supports the reliability and physical consistency of the deconvolution rather than a purely mathematical optimization. The deconvolution process demonstrated exceptional mathematical accuracy, yielding high coefficients of determination ($\:{R}^{2}$) and extremely low reduced chi-squared ($\:{\chi\:}^{2}$) values for all studied samples (BPML: $\:{\chi\:}^{2}=7.232\times\:{10}^{-7},\:{R}^{2}=0.9995;\:$BPML: Er: $\:{\chi\:}^{2}=1.622\times\:{10}^{-6},\:{R}^{2}=0.9997;$ BPML: Nd: $\:{\chi\:}^{2}=1.375\times\:{10}^{-5},\:{R}^{2}=0.9987;$ BPML: Er/Nd-1: $\:{\chi\:}^{2}=3.294\times\:{10}^{-5},\:{R}^{2}=0.9986;$ BPML: Er/Nd-2: $\:{\chi\:}^{2}=3.556\times\:{10}^{-5},\:{R}^{2}=0.9998;$ BPML: Er/Nd-3: $\:{\chi\:}^{2}=2.466\times\:{10}^{-4},\:{R}^{2}=0.9954;$). Furthermore, the confidence intervals for peak positions were found to remain within ± 2–5 cm^− 1^, indicating strong positional reliability of the resolved bands and supporting the robustness of the fitting model. The bands at 455, 501, and 552 cm^− 1^ are attributed to the modifier cations, including $\:\mathrm{P}\mathrm{b}-\mathrm{O}$ bond vibrations^41–43^, $\:\mathrm{L}\mathrm{i}-\mathrm{O}$^42, 44,45^, and $\:\mathrm{M}\mathrm{g}-\mathrm{O}$^16, 46,47^ interaction within the glass network. The absorption band observed at 618 cm^− 1^ arises from $\:\mathrm{P}\mathrm{b}-\mathrm{O}$ stretching^42,48^, overlapping with $\:\mathrm{O}-\mathrm{B}-\mathrm{O}$ bending vibration^42^. The absorption band at 675 cm^− 1^ corresponds to the bending modes of BO_3_ units^16,43,48^, whereas the 718 cm^− 1^ band is generally associated with $\:\mathrm{B}-\mathrm{O}-\mathrm{B}$ linkages between borate structural units^49–50^. The 773 cm^− 1^ band is commonly assigned to $\:\mathrm{B}-\mathrm{O}-\mathrm{B}$ bending vibrations involving linkages between $\:{\mathrm{B}\mathrm{O}}_{3}$ and $\:{\mathrm{B}\mathrm{O}}_{4}$ structural units, which is consistent with the coexistence of trigonal ($\:\mathrm{B}{\mathrm{O}}_{3}$) and tetrahedral ($\:\mathrm{B}{\mathrm{O}}_{4}$) borate units within the glass network^49^. Similarly, the bands at 827 and 877 cm^− 1^ are commonly attributed to BO_4_ stretching and diborate vibrations within the BO_4_ framework, respectively^25,50^. The band at 951 cm^− 1^ is generally assigned to $\:\mathrm{B}-\mathrm{O}$ stretching in BO_4_ units associated with di-borate groups^50^, the strong band at 1024 cm^− 1^ represents $\:\mathrm{B}-\mathrm{O}$ stretching in BO_4_ tetrahedra belonging to tri-, tetra-, and penta-borate configurations^51–52^. The band at 1104 cm^− 1^ is also consistent with the presence of penta-borate units, indicating the structural diversity of tetrahedral borate units^51^. The absorption band at 1205 cm^− 1^ is assigned to $\:\mathrm{B}-\mathrm{O}$ stretching in trigonal BO_3_ units arranged in boroxol ring structures^49–50^, and the 1268 cm^− 1^ corresponds to the stretching vibrations of isolated triangular BO_3_ units^52–53^. The peak at 1343 cm^− 1^ is attributed to the stretching vibrations of BO_3_ triangles containing non-bridging oxygens (NBOs) in meta-, pyro, and ortho-borate environments^54^, while the band at 1392 cm^− 1^ is likewise associated with similar BO_3_ environments^49^. The band at 1455 cm^− 1^ correspond to asymmetric $\:\mathrm{B}-\mathrm{O}$ stretching of BO_3_ units with NBOs^55–56^ and asymmetric stretching relaxation of trigonal BO_3_ units^57^. Table 4 illustrates the shifts in FTIR bands following the Er^3+^, Nd^3+^, and Er^3+^/Nd^3+^ incorporation. The systematic band shifts and the appearance of additional absorption features indicate that Er^3+^ and Nd^3+^ doping influences the local bonding environments within the borate glass network. Although FTIR spectroscopy provides valuable information on vibrational changes, the following structural interpretations are based on the characteristic vibrational assignments reported in the literature and should be regarded as indirect evidence of structural evolution. In the low frequency region (400–600 cm^− 1^), new bands at 412-421 and 482–493 cm^− 1^ appear, corresponding to $\:\mathrm{E}\mathrm{r}-\mathrm{O}$ and $\:\mathrm{N}\mathrm{d}-\mathrm{O}$ coordination bonds. Their systematic appearance and persistence throughout the rare earth containing glass series, together with the concurrent evolution of the neighboring low-frequency vibrational envelope, identify these features as characteristic vibrational contributions of $\:\mathrm{R}\mathrm{E}-\mathrm{O}$ coordination environments within the borate glass network, in agreement with previous reports^58–60^. To provide additional insight into the origin of these low-frequency vibrations, the characteristic $\:\mathrm{E}\mathrm{r}-\mathrm{O}$ and $\:\mathrm{N}\mathrm{d}-\mathrm{O}$ stretching frequencies were estimated using the classical diatomic harmonic oscillator approximation. This treatment serves as a first-order description of the local metal-oxygen interaction and offers a convenient framework for comparing characteristic vibrational ranges. Owing to the intrinsically disordered nature of the borate glass network, where vibrational modes arise from coupled network dynamics rather than isolated chemical bonds, the calculated frequencies are intended as qualitative reference values for local coordination environments rather than exact representations of the experimentally observed collective vibrations. The stretching frequencies of $\:\mathrm{E}\mathrm{r}-\mathrm{O}$ and $\:\mathrm{N}\mathrm{d}-\mathrm{O}$ bonds, calculated using the diatomic approximation, were found to fall within the 350–452 cm^− 1^ range (assuming force constants of 200–300 N/m and calculated reduced masses of ≈ 14.61 amu for $\:\mathrm{E}\mathrm{r}-\mathrm{O}$ and 14.40 amu for $\:\mathrm{N}\mathrm{d}-\mathrm{O}$), which lies within the spectral region where the experimentally resolved absorption bands are observed and therefore provides qualitative support for their assignment to rare earth oxygen coordination. Concurrent red shifts of $\:\mathrm{P}\mathrm{b}-\mathrm{O}$ and $\:\mathrm{O}-\mathrm{B}-\mathrm{O}$ bending modes were observed, as listed in Table 4, indicating bond weakening and elongation due to substitution of Pb^2+^ by larger rare earth cations. This is quantitatively evidenced by a decrease in the calculated force constants (* k*), confirming the reduction in the bond’s restorative force upon the introduction of RE^3+^ ions. In the mid-frequency region (650–900 cm^− 1^), several structural rearrangements are observed, associated with BO_3_/BO_4_ vibrations and $\:\mathrm{B}-\mathrm{O}-\mathrm{B}$ linkages. The disappearance of the 675 cm^− 1^ bands together the downward shift of the 718 cm^− 1^ bands (to 696–700 cm^− 1^) may indicate modifications in the relative populations of BO_3_ and BO_4_ units and slight variations in $\:\mathrm{B}-\mathrm{O}-\mathrm{B}$ bond angles. The shift from 718 to 700 cm^− 1^ corresponds to a reduction in the force constant from 208 N/m to 196 N/m, which is compatible with a relaxation of the borate bridging network. The enhanced or blue-shifted 773 cm^− 1^ band (up to 798 cm^− 1^) suggests stronger coupling between BO_3_ and BO_4_ species, while the shifts of the 827 cm^− 1^ and 877 cm^− 1^ bands to 802 cm^− 1^ and 849 cm^− 1^, respectively, suggests changes in the local BO_4_ environments and possible interactions between Er^3+^/Nd^3+^ ions and neighboring borate structural units. These transformations signify partial network depolymerization and creation of new cross-links between BO_3_ and BO_4_ units. In the high frequency region (900-1200) cm^− 1^, corresponding to the asymmetric $\:\mathrm{B}-\mathrm{O}$ stretching vibrations of BO_4_ tetrahedra, the bands at 951 cm^− 1^ and 1024 cm^− 1^ exhibit a blue shift in BPML: Er and BPML: Er/Nd-3. These shifts are consistent with strengthened $\:\mathrm{B}-\mathrm{O}$ bonds resulting from the relatively high ionic field strength of Er^3+^, which promotes stronger local $\:\mathrm{R}\mathrm{E}-\mathrm{O}$ interactions and enhances the rigidity of the surrounding borate environment. The observed bule shift is consistent with an increase in the effective local bond stiffness and stronger $\:\mathrm{B}-\mathrm{O}$ interactions within the $\:{\mathrm{B}\mathrm{O}}_{4}$ structural units. Within the amorphous borate glass network, these changes are interpreted in terms of relative variations in local bonding environments inferred from the vibrational spectra. Conversely, BPML: Nd, BPML: Er/Nd-1, and BPML: Er/Nd-2 display red shifts, suggesting weakened $\:\mathrm{B}-\mathrm{O}$ bonds. The weakening is most pronounced in BPML: Nd, where the 951 cm^− 1^ band shifts to 929 cm^− 1^ and the 1024 cm^− 1^ band disappears entirely. The calculated force constant for the 951 cm^− 1^ band decreases from 365 to 348 N/m, providing a semi-quantitative indication of reduced bond stiffness that is in agreement with experimentally observed spectral red shift and the inferred increase in average $\:\mathrm{B}-\mathrm{O}$ bond strength. The appearance of additional bands within the 880–906 cm^− 1^ and 1062-1076 cm^− 1^ ranges may also indicate changes in the local coordination environment around the RE^3+^ ions and borate structural units. Although RE^3+^ are generally regarded as network modifiers in borate glasses, their relatively high ionic field strength can influence the local coordination environment through strong $\:\mathrm{R}\mathrm{E}-\mathrm{O}$ interactions these interactions are expected to modify the connectivity and distribution of neighboring borate structural units, leading to local structural rearrangements. In the upper frequency region (1200-1600) cm^− 1^, dominated by $\:\mathrm{B}-\mathrm{O}$ stretching in BO_3_ units and NBO-related modes, the progressive blue shift of 1268 cm^− 1^ band to 1309 cm^− 1^ and the splitting of the 1343–1392 cm^− 1^, as listed in Table 4, suggest modifications in the local bonding environment and possible changes in the distribution of non-bridging oxygens. This indicates enhanced covalency and reduced NBO concentration through $\:\mathrm{R}\mathrm{E}-\mathrm{O}-\mathrm{B}$ bond formation. Applying the harmonic oscillator model, the force constant was found to change from approximately 694 to 692 N/m, reflecting only a slight variation in bond stiffness within the $\:{\mathrm{B}\mathrm{O}}_{3}$ structural units. Such estimated force constants provide a useful comparative descriptor for monitoring relative changes in local bonding environments across the investigated glass composition, while the observed FTIR bands remain collective vibrational responses of the disordered borate network. The blue shift of asymmetric BO_3_ stretching bands 1455 cm^− 1^ may reflect $\:\mathrm{B}-\mathrm{O}$ bond strengthening and polarization under the influence of highly polarizing rare-earth ions.

**Table 3:** Oscillation modes of the considered BPML glass series and the corresponding FTIR positions.

Peak position (cm^− 1^)	Oscillation mode
455	Bending vibrations of the $\:\mathrm{O}-\mathrm{B}-\mathrm{O}$ bond, overlapping with $\:\mathrm{P}\mathrm{b}-\mathrm{O}$ bond vibrations, and also vibrational modes associated with Mg^2+^ ions
501,552	Vibrational modes associated with modifier cations, particularly Li^+^ ions.
618	Vibrational modes associated with $\:\mathrm{P}\mathrm{b}-\mathrm{O}$ bonds, overlapping with $\:\mathrm{O}-\mathrm{B}-\mathrm{O}$ bending vibration
675	BO_3_ bending vibrations.
718	$\:\mathrm{B}-\mathrm{O}-\mathrm{B}$ bending vibrations.
773	$\:{\mathrm{B}}_{3}\mathrm{O}-\mathrm{O}-\mathrm{B}{\mathrm{O}}_{4}$ bending vibrations.
827	Stretching of tetrahedral BO_4_ groups.
877	Stretching vibrations of tetrahedral borate groups such as diborate in BO_3_.
951	The $\:\mathrm{B}-\mathrm{O}$ stretching vibrations in tetrahedral BO_4_ in di-borate.
1024	Stretching vibrations of $\:\mathrm{B}-\mathrm{O}$ in BO_4_ units from tri- tetra- and penta-borate groups.
1104	Stretching vibrations of tetrahedral borate groups such as penta-borate groups.
1205	$\:\mathrm{B}-\mathrm{O}$ stretching vibration of trigonal BO_3_ units in boroxol rings.
1268	Ascribed to the stretching vibration of triangular BO_3_ units.
1343	Stretching vibrations of BO_3_ triangles with non-bridging oxygens (NBOs) (stretching of $\:\mathrm{B}-\mathrm{O}$ in meta-borate, pyro-borate, and ortho-borate groups of BO_3_).
1392	$\:\mathrm{B}-\mathrm{O}$ stretching vibrations of BO_3_ units in meta-, pyro- and ortho-borate groups.
1455	Asymmetric $\:\mathrm{B}-\mathrm{O}-$ stretching vibration of BO_3_ triangles containing non-bridging oxygens (NBOs).

**Table 4:** The shifts in FTIR bands of the (BPML) network with the introducing Er, Nd, and Er / Nd.

New band	BPML	BPML: Er	BPML: Nd	BPML: Er/Nd-1	BPML: Er/Nd-2	BPML: Er/Nd-3
	-	412	404	403	415	421
	455	455	466	451	456	-
New band	-	482	-	481	476	493
	501	513	513	519	523	534
	552	533	522	-	-	-
	618	605	593	612	604	597
	675	-	-	-	-	-
	718	696	700	698	697	699
	773	-	-	798	794	-
	827	802	825	823	833	816
	877	866	866	849	845	876
New band	-	906	896	880	882	889
	951	959	929	947	942	961
	1024	1029	-	1024	1024	1026
New band	-	1069	1062	1076	-	1065
	1104	1119	1119	1116	1103	1108
	1205	1230	1210	1217	1229	1206
	1268	1285	1296	1309	1312	1309
	1343	1347	1353	1356	1364	1348
	1392	-	-	1402	1390	-
	1455	1473	1481	1478	1484	1470

### Thermal properties

**Fig. 4:**
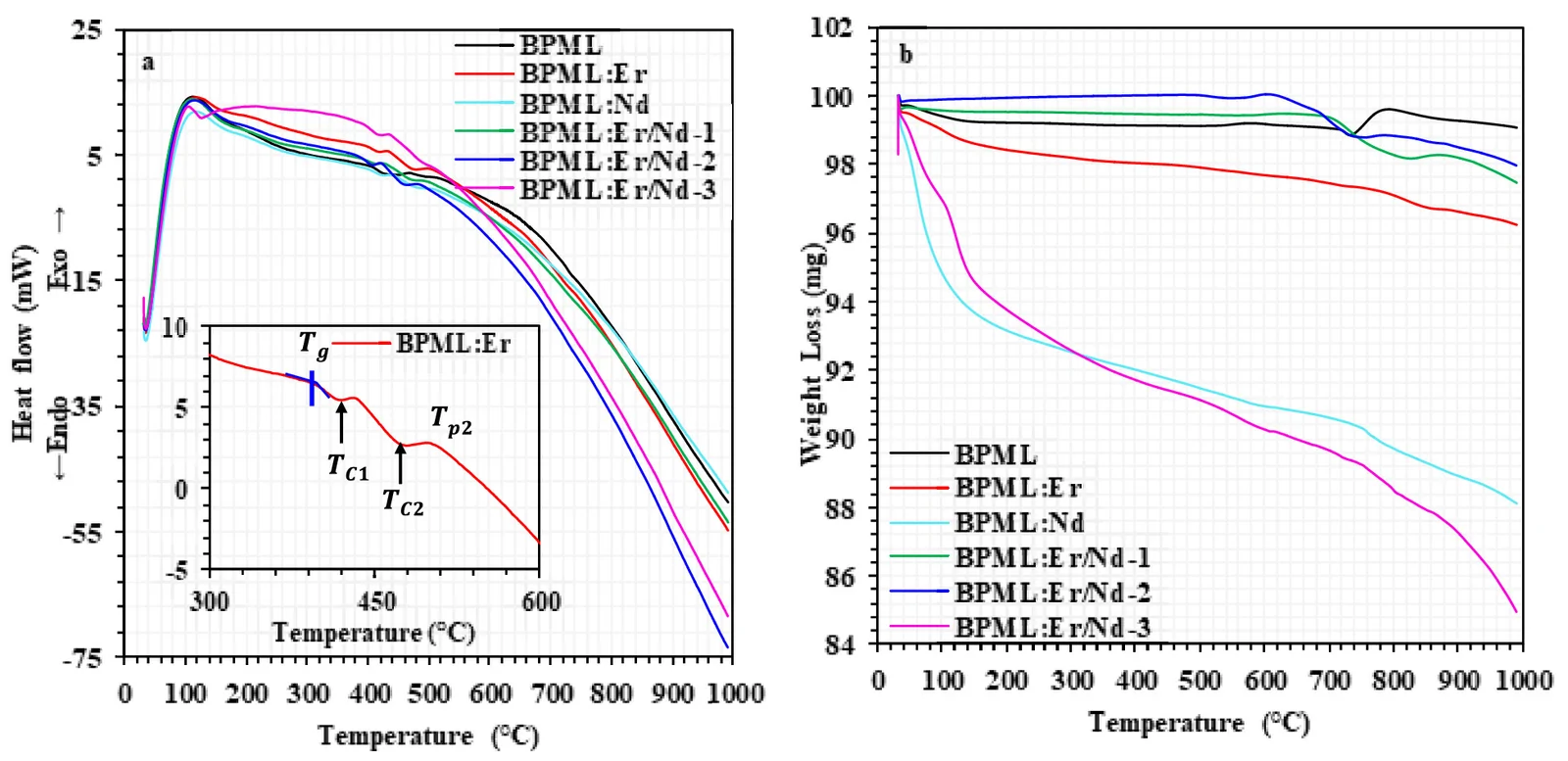
The measured thermal profile of the BPML and BPML: RE^3+^ glass series using the (a) DSC and (b) TGA.

The thermal performance of the BPML glass series, as revealed by DSC and TGA analyses Fig. 4, reflects the intricate structural modifications induced by RE-doping, as previously discussed. The DSC curve shown in Fig. 4a estimate the thermal parameters, glass transition temperature ($$\:{T}_{g}$$) and crystallization onset and peak temperatures ($$\:{T}_{c1}$$& $$\:{T}_{c2}$$ and $$\:{T}_{p1}$$ & $$\:{T}_{p2}$$). The $$\:{T}_{g}$$ of the base glass (BPML) is observed at 405 °C, with $$\:{T}_{c1}=453$$ °C, $$\:{T}_{c2}=493$$ °C, $$\:{T}_{p1}=468$$ °C, and $$\:{T}_{p2}=511$$ °C, indicating a stable and well-connected borate network structure^61^. Upon doping with Er^3+^ (BPML: Er), the glass transition and crystallization temperatures were decreased^61–62^, as listed in Table 5. Although density and FTIR results confirm a more compact local environment and an increased BO_4_/BO_3_ ratio, the $$\:{T}_{g}$$ depression must be interpreted through the thermodynamic evolution of the network. In accordance with the Adam-Gibbs model ($$\:{T}_{g}\propto\:\raisebox{1ex}{$1$}\!\left/\:\!\raisebox{-1ex}{${S}_{c}$}\right.$$), the decline in $$\:{T}_{g}$$ reflects an expanded configurational entropy ($$\:{S}_{c}$$). This is attributed to the introduction of Er^3+^ ions, which diversify the local bonding manifold and create heterogeneous $$\:\mathrm{E}\mathrm{r}-\mathrm{O}-\mathrm{B}$$ environments that facilitate cooperative rearrangements. Such structural complexity lowers the activation barrier for structural relaxation, facilitating segmental mobility through entropic gains that override the effects on increased physical packing^63–64^. Crucially, this structural divergence under thermal excitation reveals a decoupling between physical compactness and thermal stability; while the higher concentration of BO_4_ tetrahedra rigidifies the matrix at room temperature, the multi-component nature of the RE-modified network offers more degrees of freedom at high temperatures, hence lowering $$\:{T}_{g}$$. The high $$\:{T}_{p2}$$ value supports this, indicating that Er^3+^ strengthens the glass network against crystallization at elevated temperatures, consistent with its high field strength and strong bonding. In contrast, Nd^3+^ doping (BPML: Nd) exhibits slightly higher glass transition and crystallization temperatures compared to BPML: Er, as listed in Table 5. This trend aligns with the structural data, where Nd^3+^ induces less compactness due to its larger ionic radius and weaker field strength^21,65–66^. The higher $$\:{T}_{g}$$ of BPML: Nd arises from its lower packing efficiency which, in this host, restricts the configurational degrees of freedom ($$\:{S}_{c}$$), thereby requiring higher thermal energy to trigger the glass transition. Hence, while the BPML: Nd glass transitions at a slightly higher temperature, its network is less tightly connected and more open compared with BPML: Er^22,67^. This lower packing density and structural openness create a loose framework that structurally resists short-range cooperative relaxation up to a slightly higher thermal threshold, although it possesses lower overall thermodynamic stability. Moreover, the co-doped samples show further reductions in $$\:{T}_{g}$$. This is consistent with the structural evidence that mixed doping introduces a more complex structural rearrangement, further increasing the configurational entropy through the proliferation of diverse bond angles and lengths. The decrease in $$\:{T}_{g}$$ for BPML: Er/Nd-1 and BPML: Er/Nd-2 suggests that the coexistence of Er^3+^ (promoting compactness) and Nd^3+^ (enhancing flexibility) produces a more dynamically stable yet tightly packed structure. Among them, BPML: Er/Nd-2 exhibits the most favorable thermal profile, with moderate $$\:{T}_{g}$$ and the highest $$\:{T}_{p2}$$. This implies an optimal structural balance. In BPML: Er/Nd-3 where the disappearance of $$\:{T}_{c2}$$ and $$\:{T}_{p2}$$ reflects that excessive rare earth loading disrupts network uniformity. According to the classical nucleation consideration, the disappearance of these crystallization events may be associated with suppression of homogeneous nucleation and crystal growth kinetics due to increased structural disorder and restricted ionic diffusion within the highly modified glass network. In addition, the coexistence of multiple rare-earth environments at high dopant concentrations may promote local compositional heterogeneity or amorphous phase-separated regions, thereby preventing the formation of distinct secondary crystallization peaks. That leads to inhomogeneous regions and reduced thermal stability. The highly doped network becomes more disordered, hindering secondary crystallization and consistent with the broader FTIR features; thus, indicating structural distortion at high RE^3+^ concentrations. Such behavior is also consistent with the disappearance of well-defined crystallization pathways in highly heterogeneous glass systems, where nucleation becomes kinetically unfavorable. Figure 4b shows the TGA results across the temperature range up to ~ 990 °C. The results reveal distinct multistage mass change behavior, as shown in, providing complementary insight into thermal decomposition, volatilization, and structural rearrangement. For (BPML), the mass loss from 44 to 148 °C (99.7–99.2%) is likely corresponding to the evaporation of physically adsorbed water and residual hydroxyl groups. The broad thermal stability plateau from 149 to 528 °C (99.2–99.1%) indicates a thermally resilient network with minimal structural breakdown. Interestingly, the slight mass increases observed at 529–583 °C and a gain at 733–800 °C suggest recrystallization or oxidation effects, possibly associated with redox activity of Pb^2+^ or structural rearrangements involving BO_3_/BO_4_ interconversion. The final drop from 801 to 990 °C reflects partial network degeneration. A more continuous and pronounced mass loss (99.5–96.2%) in BPML: Er glass across the entire range, without clear plateaus. This behavior suggests a progressive breakdown of the glass network upon heating. In BPML: Nd, an early weight loss (43–143 °C, 99→93.7%), likely from less compact Nd^3+^-dominated structures allowing faster release of bound water or $$\:{\mathrm{O}\mathrm{H}}^{-}$$ groups. Structurally, the less compact and more open network configuration identified in BPML: Nd facilitates the rapid diffusion and outgassing of volatile hydroxyl species at lower thermal ranges. The steady decline to 88.1% by 990 °C reflects ongoing network disruption due to weaker Nd^3+^-O interactions. Mixed doping, particularly in BPML: Er/Nd-2, yields complex TGA behavior, including regions of weight gain due to transient structural reorganization or oxidation, further suggesting an optimal balance between network flexibility and compactness. Notably, BPML: Er/Nd-3 exhibited a significant and continuous mass loss (from 99.3% to 85.0%), confirming that high dopant concentrations destabilize the network, resulting in reduced thermal resistance.

**Table 5:** Thermal parameters (°C) of the considered BPML and BPML: RE^3+^ glass series.

Glass sample code	$\:{\boldsymbol{T}}_{\boldsymbol{g}}$	$\:{\boldsymbol{T}}_{\boldsymbol{C}1}$	$\:{\boldsymbol{T}}_{\boldsymbol{P}1}$	$\:{\boldsymbol{T}}_{\boldsymbol{C}2}$	$\:{\boldsymbol{T}}_{\boldsymbol{P}2}$
BPML	408	451	469	495	512
BPML: Er	398	418	428	478	498
BPML: Nd	404	426	438	480	507
BPML: Er/Nd-1	392	413	427	473	494
BPML: Er/Nd-2	392	405	422	463	478
BPML: Er/Nd-3	380	418	436	ــ	ــ

### Theoretical mechanical properties

From Table 6 it is evident that there are systematic increases in the predicted mechanical features of the sensitized borate-based glass system with the inclusion of Er^3+^, Nd^3+^, and Er^3+^/Nd^3+^. BPML: Er exhibited the highest predicted improvement among the investigated samples, with observed increases across all key mechanical parameters. These enhancements are directly correlated with the structural modifications identified via density, density related parameters, and FTIR analyses. Specially, the substantial increase in the predicted elastic moduli (Young’s bulk, and shear moduli) and Vickers hardness is fundamentally rooted in the higher dissociation energy od $\:\mathrm{R}\mathrm{E}-\mathrm{O}$ bonds ($\:\mathrm{E}\mathrm{r}-\mathrm{O}$ ≈ 611 kJ/mol) compared to the host modifier bonds ($\:\mathrm{P}\mathrm{b}-\mathrm{O}$ ≈ 378 kJ/mol). The integration of Er^3+^ and Nd^3+^ ions strengthens the total network energy of the glass structure. Furthermore, the enhanced BO_4_/BO_3_ ratio derived from the FTIR analysis indicates increased network connectivity, whereas the higher OPD and $\:{V}_{t}$ values signify more efficient atomic packing and structural densification. Therefore, the improved mechanical performance arises from the combined effects of network polymerization, bond strengthening, and enhanced packing efficiency. The substantial transformation of threefold-coordinated BO_3_ units into highly crosslinked fourfold-coordinated BO_4_ tetrahedra acts as a structural polymerization tool, creating a rigid three-dimensional network that effectively resists external shear stresses and mechanical indentation. These findings are consistent with previous reported by Abo-Mosallam et al.^68^ and Ngaram et al.^69^ on Er^3+^-doped borate glasses, where the incorporation of Er^3+^ increased the elastic moduli and hardness through stronger $\:\mathrm{R}\mathrm{E}-\mathrm{O}$ bonding, improving atomic packing, and enhanced network connectivity. While the impacts of Nd^3+^ doping (BPML: Nd) were slightly less pronounced than those of Er^3+^, notable improvements in the predicted mechanical performance are still observed. These are likely due to partial network reinforcement, although limited by the larger ionic radius and lower field strength of Nd^3+^, resulting in weaker $\:\mathrm{N}\mathrm{d}-\mathrm{O}$ bonding. For the co-doped glass samples (BPML: Er/Nd-1, BPML: Er/Nd-2, and BPML: Er/Nd-3), the predicted mechanical properties remained consistently high, indicating that the combined incorporation of Er^3+^ and Nd^3+^ effectively preserves the enhanced stiffness and predicted resistance to deformation achieved through rare earth modification of the glass network. A similar enhancement in the predicated elastic properties with Nd^3+^ incorporation has been reported by Abouhaswa et al.^70^, although an opposite trend was observed by Singh et al.^71^, who attributed the reduction in elastic moduli to network depolymerization and increased non-bridging oxygen formation. This comparison suggests that the mechanical response of Nd^3+^ is strongly dependent on the host glass composition and the structural role of rare earth ions. Notably, Poisson’s ratio ($\:\sigma\:$) remains nearly constant across all samples, varying only from 0.252 to 0.255. According to the theory of elastic properties of glasses proposed by Bridge and Higzay^72^, Poisson’s ratio is fundamentally dictated by the cross-link density and the spatial dimensionality of the glass network. To provide a deeper theoretical insight, this behavior can be rationalized using the Philips-Thorpe constraint counting model^73^, which correlates the structural rigidity and mechanical constraints within the network connectivity. In the present borate glass system, the structural transition involves the conversion of threefold coordinated borate units (BO_3_) into fourfold coordinated units (BO_4_). Although the fraction of BO_4_ units (N_4_) increases slightly from 0.485 to 0.543 (corresponding to a minor shift in the N_4_/N_3_ ratio from 0.941 to 1.188), this structural modification is relatively subtle. Within the framework of the Philips-Thorpe model, such small change in the population of tetrahedral constraints does not trigger a fundamental topological phase transition or a major alteration in the overall cross-link density of the glass network. Consequently, the core spatial network dimensionality remains closely preserved, which is theoretically consistent with the highly stable values of $\:\sigma\:$. This preservation of spatial dimensionality underscores that the reinforcement of the glass network upon RE-incorporation occurs primarily through bond-stiffening. This correlation indicates that the glass samples exhibited higher predicted elastic moduli and hardness without substantially increasing their capacity to absorb strain before failure. In addition, rare earth incorporation modifies the local network environment through $\:\mathrm{R}\mathrm{E}-\mathrm{O}$ interactions, promoting a more compact glass structure with improved atomic packing efficiency. Consequently, the enhanced mechanical performance results from the combined effects of network strengthening, structural densification, and increased packing efficiency. Furthermore, the consistent increase in hardness that is observed in all doped samples attributed to the combined effects of $\:\mathrm{R}\mathrm{E}-\mathrm{O}$ bonding, increased $\:{BO}_{4}$ formation, improved atomic packing efficiency, and the development of a denser and more interconnected glass network, all of which contribute to improved resistance to surface deformation.

Finally, the obtained values of the mechanical parameters should be interpreted primarily in a comparative rather than absolute manner due to the complex multicomponent nature of the present oxyfluoride glass system. Moreover, the Makishima-Mackenzie model does not explicitly account for local structural heterogeneity, mixed-cation interactions, or BO_4_/BO_3_ related network connectivity changes. Nevertheless, the calculated trends remain in good agreement with the structural modifications inferred from density and FTIR analyses, as well as with previously reported behavior for related rare-earth-containing borate glass systems^74–75^.

**Table 6:** The mechanical properties of the BPML and BPML: RE^3+^ glass samples.

Glass sample code	E (GPa)	K (GPa)	G (GPa)	L (GPa)	$\:\boldsymbol{\upsigma\:}$	H (GPa)
BPML	45.915	30.846	18.338	55.297	0.252	3.033
BPML: Er	47.459	32.300	18.906	57.508	0.255	3.087
BPML: Nd	47.310	32.115	18.856	57.257	0.254	3.086
BPML: Er/Nd-1	47.391	32.216	18.884	57.394	0.255	3.086
BPML: Er/Nd-2	47.357	32.175	18.872	57.338	0.255	3.086
BPML: Er/Nd-3	47.425	32.258	18.895	57.451	0.255	3.087

### Optical properties

**Fig. 5:**
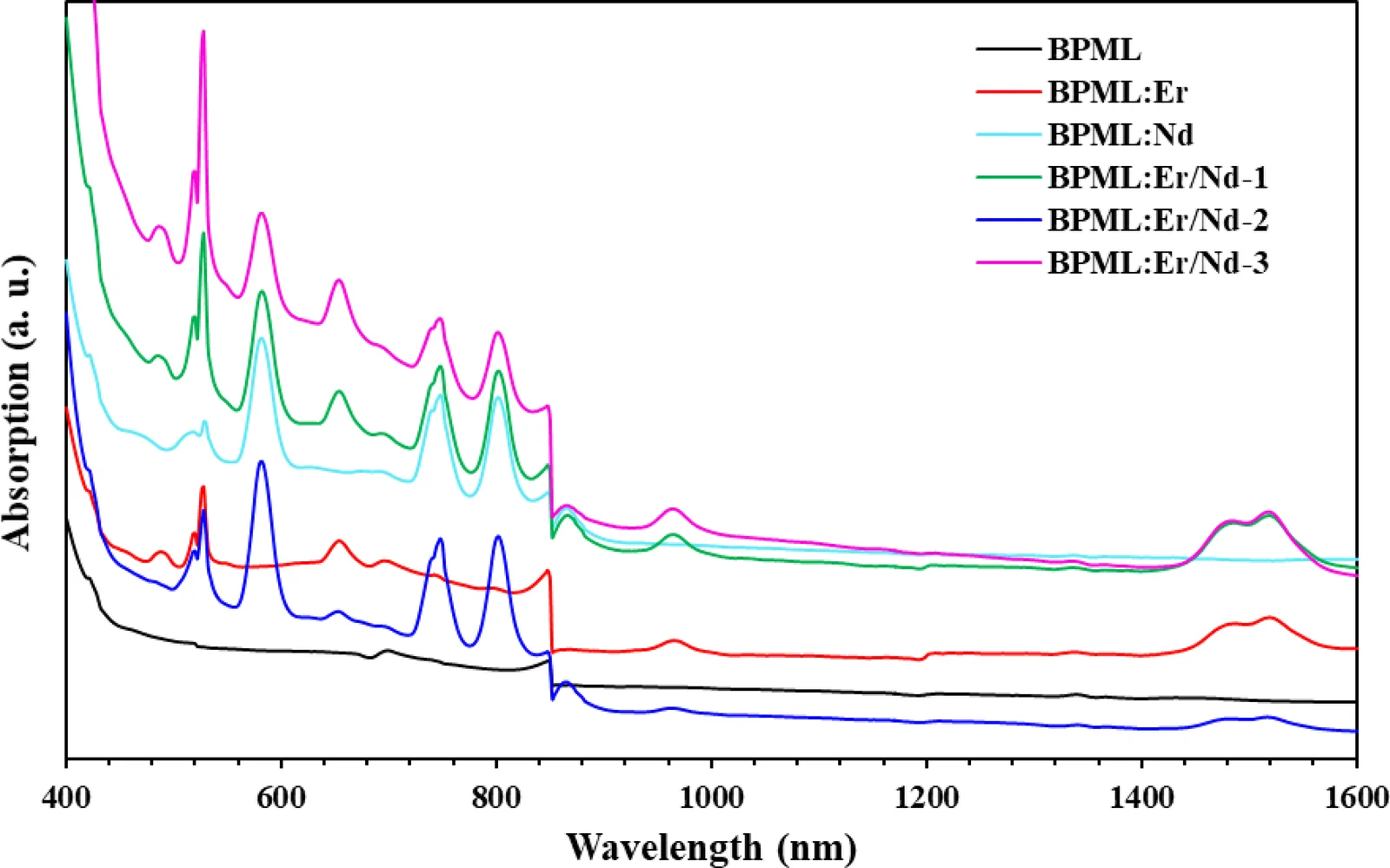
The recorded absorption spectra of the synthesized glass samples.

Figure 5 illustrates the optical absorption spectra of the synthesized BPML: RE^3+^ glass series. No absorption bands were observed in the undoped BPML glass, confirming that all observed absorption features are present in the doped and co-doped glass samples originate from the electronic transitions of Er^3+^ and Nd^3+^ ions. For the BPML: Er, distinct absorption bands were recorded at 488, 520, 527, 654, 702, 801, 966, 1496, and 1518 nm. The transitions at 488, 801, and 966 nm correspond to excitation from the Er^3+^ ground state (^4^I_15/2_) to the ^4^F_7/2_, ^4^I_9/2_, and ^4^I_11/2_ excited states, respectively, while the paired bands at 520 & 527, 654 & 702, and 1469 & 1518 nm result from the crystal-field splitting of the ^2^H_11/2_, ^4^F_9/2_, and ^4^I_13/2_ manifolds. The observed splitting arises from the interaction between the Er^3+^ 4f electrons and the asymmetric electric field produced by surrounding oxygen ligands in the glass network. Although the 4f orbitals are partially shielded by outer 5s and 5p electrons, this crystal-field interaction is sufficient to lift the degeneracy of energy levels, reflecting variations in local structure and site asymmetry around the Er^3+^ ions. The strong absorption band at 527 nm and the weaker, nascent band at 520 nm correspond to transitions from ^4^I_15/2_→^2^H_11/2_, with their differing intensities indicating the presence of non-equivalent Er^3+^ sites in the glass system. To provide a rigorous, quantitative justification for these intensity variations and local environment features, a comprehensive Judd-Ofelt (J-O) analysis was performed to determine the phenomenological intensity parameters ($\:{{\Omega\:}}_{2},\:{{\Omega\:}}_{4},\:{{\Omega\:}}_{6}$). As summarized in Table 7, the quality of the least-squares fitting is statistically robust, yielding coefficient of determination ($\:{R}^{2}$) values exceeding 0.99 across all investigated samples, thereby ensuring the physical reliability of the extracted parameters. The $\:{{\Omega\:}}_{2}$ parameter provides valuable insight into the local crystal-field environment surrounding rare earth ions, reflecting variations in site symmetry and the strength of rare earth oxygen interactions. Its magnitude is governed by the local coordination characteristics, transition probabilities, fitting conditions, and host glass composition, and therefore serves as a sensitive spectroscopic parameter for evaluating structural changes around the rare earth sites. For the single doped BPML: Er sample, $\:{{\Omega\:}}_{2}$ achieves its maximum value ($\:4.85\times\:{10}^{-20}$ cm^2^), suggesting a more strongly perturbed local environment around Er^3+^ ions and enhanced interaction with the surrounding oxygen ligands. This behavior is consistent with the dominance of the split 527 nm band, which reflects increased sensitivity of the Er^3+^ electronic transitions to local crystal-field environment. Upon co-doping with Nd^3+^ ions (BPML: Er/Nd-1, BPML: Er/Nd-2, and BPML: Er/Nd-3), a systematic decrease in $\:{{\Omega\:}}_{2}$ down to $\:3.976\times\:{10}^{-20}$ cm^2^ is observed. The reduction in $\:{{\Omega\:}}_{2}$ suggests a modification of the local coordination environment surrounding the Er^3+^ ions and may be associated with changes in the crystal-field distribution induced by Nd^3+^ incorporation. Within the intrinsically disordered borate glass network, the Judd-Ofelt intensity parameters $\:{{\Omega\:}}_{2}$ embodies the combined contributions of local coordination geometry, crystal-field distribution, rare earth site redistribution, concentration-dependent ion-ion interactions, and possible local clustering of Er^3+^ and Nd^3+^ ions. Accordingly, the observed variation in $\:{{\Omega\:}}_{2}$ reflects the overall evolution of the local structural and spectroscopic environment accompanying rare earth incorporation into the glass network. Furthermore, the calculated total radiative transition probability ($\:{A}_{total}$) for the ^2^H_11/2_ → ^4^I_15/2_ transition decreases from 22,450 to 17,200 s^− 1^, providing a solid theoretical confirmation for the observed intensity variation and demonstrating that the radiative efficiency of the Er^3+^ site is inherently coupled to the Co-doped concentration and subsequent host network rearrangements. In the BPML: Nd glass, distinct absorption bands are observed at 516, 530, 582, 748, 802, 848, and 866 nm. The paired bands at 516 & 530 nm and 848 & 866 nm originate from transition from the Nd^3+^ ground state (^4^I_9/2_) to the crystal-field-split ^4^G_7/2_ and ^4^F_3/2_ levels, respectively. The bands at 582, 748, and 802 correspond to transitions from ^4^I_9/2_ to the ^4^G_5/2_, ^4^F_7/2_, and ^4^F_5/2_ excited states. The observed splitting within the ^4^G_7/2_ and ^4^F_3/2_ manifolds reflects the influence of the asymmetric crystal field produced by surrounding oxygen ligands in the glass matrix, which perturbs the Nd^3+^ 4f energy levels. As for the co-doped samples (BPML: Er/Nd-1, BPML: Er/Nd-2, and BPML: Er/Nd-3), the optical absorption spectra display characteristic transitions of both Er^3+^ and Nd^3+^ ions, confirming the successful incorporation of both rare earth samples into the BPML glass series. The spectra reveal a superposition of the well-defined absorption bands corresponding to Er^3+^ and Nd^3+^ transitions, indicating that both ions retain their individual spectroscopic identities without significant spectral interference or energy-level distortion. This coexistence of Er^3+^ and Nd^3+^ absorption features suggests that the glass network provides suitable sites for both ions, enabling potential energy transfer interactions between them.

**Table 7:** Judd-Ofelt intensity parameters of BPML: RE glass series.

Sample code	$\:{\boldsymbol{\Omega\:}}_{2}\times\:{10}^{-20}$ cm^2^	$\:{\boldsymbol{\Omega\:}}_{4}\times\:{10}^{-20}$ cm^2^	$\:{\boldsymbol{\Omega\:}}_{6}\times\:{10}^{-20}$ cm^2^
BPML: Er	4.852	0.937	0.387
BPML: Nd	1.625	0.254	0.207
BPML: Er/Nd-1	4.296	0.184	9.937
BPML: Er/Nd-2	4.111	0.180	0.148
BPML: Er/Nd-3	3.976	0.141	0.122

### Photoluminescence

**Fig. 6:**
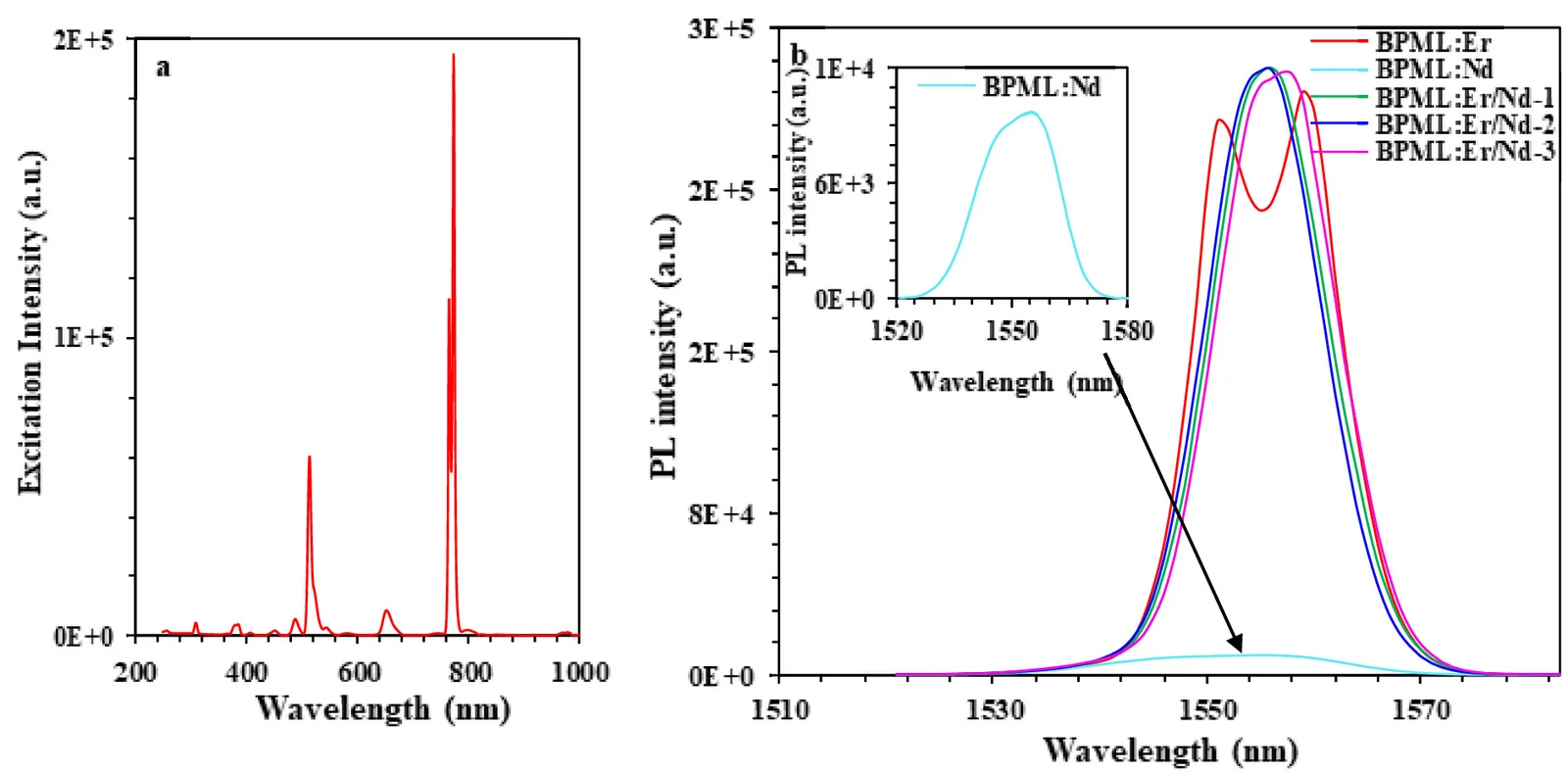
**a**) Excitation spectrum and **b**) the emitted wavelengths of the synthesized BPML: RE^3+^ glass series.

**Fig. 7:**
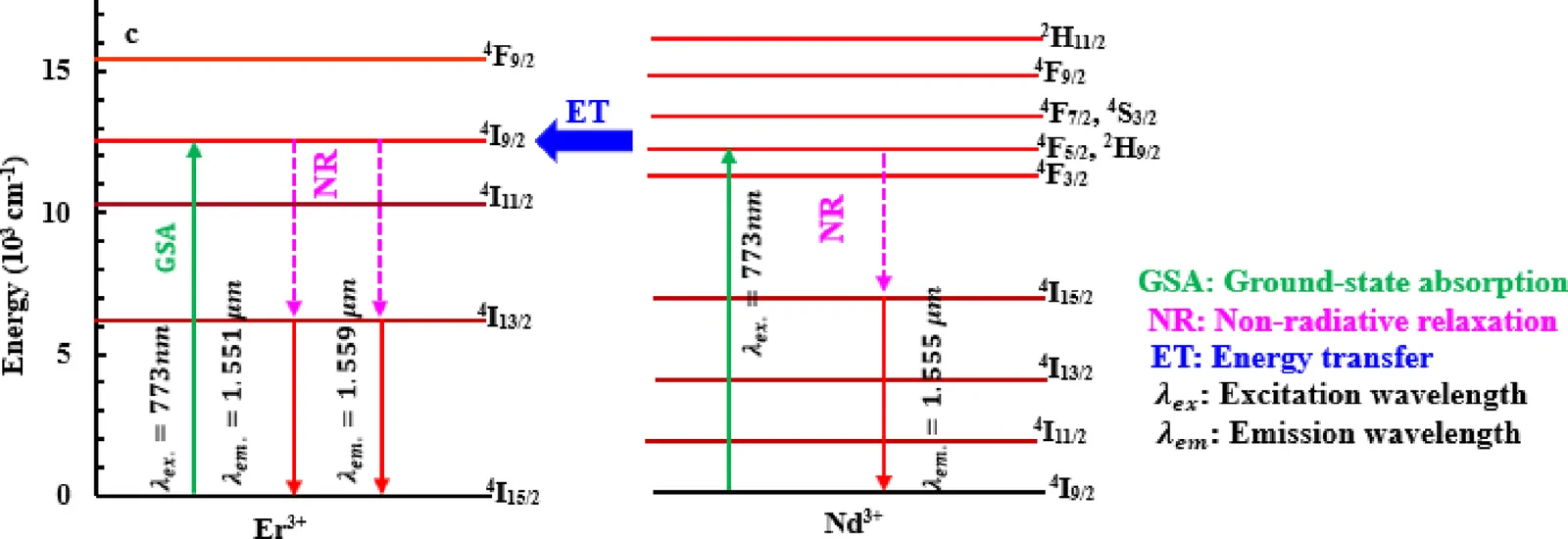
The probable transitions in Er^3+^ and Nd^3+^ ions and Er^3+^/ Nd^3+^ energy transfer.

The excitation spectrum shown in Fig. 6a reveals several resonances capable of driving emission at 1.55 μm. The excitation performed at 773 nm produces the most efficient response and has been therefore chosen to optimize the 1.55 μm emission intensity. Figure 6b illustrated the photoluminescence (PL) spectra that reveals distinct emission behaviors arising from the presence and interaction of Er^3+^ and Nd^3+^ ions in the synthesized glass samples under 773 nm excitation. The base BPML sample exhibits no detectable emission, confirming the absence of active luminescent centers in the host matrix. In contrast, the BPML: Er glass displays two pronounced emission peaks at 1.551 and 1.559 μm, corresponding to the ^4^I_13/2_ → ^4^I_15/2_ transition of Er^3+^. These dual features originate from Stark splitting of the Er^3+^ energy levels within the glass network. Upon 773 nm excitation, Er^3+^ ions are promoted to the ^4^I_9/2_ state via ground-state absorption (GSA), as shown in Fig. 7, and subsequently undergo fast non-radiative (NR) relaxation through the ^4^I_9/2_ intermediate state to the ^4^I_13/2_ metastable level, from which the characteristic 1.55 μm emission occurs. For the BPML: Nd sample, in contrast, exhibits only weak emission near 1.555 μm, which may be associated with weak Stark-broadened Nd^3+^-related emission features and local phonon-assisted processes within the glass network. However, the significant lower intensity compared with the Er-containing samples indicates that Nd^3+^ does not act as the primary luminescent center responsible for the 1.55 μm emission. The low intensity arises from inefficient Nd^3+^ excitation at 773 nm and intrinsically low radiative probability of this transition. Remarkably, the co-doped samples (BPML: Er/Nd-1, BPML: Er/Nd-2, and BPML: Er/Nd-3) show a single intensified emission band centered at 1.555, 1.556, and 1.558 μm, respectively. Although the emission originates primarily from Er^3+^ ions, its intensity is markedly enhanced by energy transfer (ET) from Nd^3+^ ions. As illustrated in Fig. 7, Nd^3+^ ions initially absorb the 773 nm excitation through the ^4^I_9/2_→ (^4^F_5/2_, ^2^H_9/2_) transition. The excited Nd^3+^ ions subsequently undergo rapid multiphonon non-radiative relaxation before transferring their excitation energy non-radiatively to neighboring Er^3+^ ions. This ET process effectively populates the metastable ^4^I_13/2_ level of Er^3+^, thereby increasing the population density of the emitting state and consequently enhancing the radiative ^4^I_13/2_→^4^I_15/2_ transition responsible for 1.55 μm emission. The proposed energy-transfer mechanism is consistent with previous investigation on Nd^3+^/Er^3+^ co-doped systems. Katta et al.^76^. demonstrated that cooperative Nd^3+^→Er^3+^ energy transfer substantially enhances the population of the Er^3+ 4^I_13/2_ level, leading to stronger near-infrared emission and high energy-transfer efficiency. Likewise, Ding et al.^23^ reported that efficient interionic energy transfer among rare-earth ions plays a key role in enhancing broadband near-infrared luminescence in co-doped tellurite glasses. A similar enhancement of the Er^3+^ 1.55 μm emission through improved energy-transfer efficiency has also been reported by Hu et al.^77^ in Er-based co-doped systems. The agreement between the present results and these earlier studies further supports the proposed ET mechanism in the investigated BPML glasses. The gradual shift from 1.551 μm (in BPML: Er) to 1.558 μm (in BPML: Er/Nd-3) remains relatively small and may reflect subtle modifications in the local glass environment, including structural disorder and variations in RE^3+^ ion distribution. Therefore, the observed shift is discussed cautiously without direct quantitative crystal-field assignment. The enhancement in the emission wavelength is further supported by quantitative analysis of the donor-acceptor interaction. The calculated spectral overlap integral (* J*) was found to be $\:1.68\times\:{10}^{-23}$ cm^6^/mol, yielding a critical transfer distance ($\:{R}_{0}$) of 6.42 Å. Such a short critical distance indicates that Nd^3+^ donor ions and Er^3+^ acceptor ions are sufficiently close for efficient non-radiative dipole-dipole interaction. Consequently, the calculated donor-acceptor separation provides quantitative evidence that the observed increase in the emission intensity originates from efficient Nd^3+^→Er^3+^ energy transfer rather than from independent excitation of Er^3+^ ions alone.

The emission spectra of the synthesized BPML: RE^3+^ glass samples were analyzed in the frequency domain to determine the center frequency ($\:{\mathrm{f}}_{0}$), spectra bandwidth $\:(\varDelta\:\mathrm{F})$, and corresponding quality factor

16 $$\:{\mathrm{Q}}_{\mathrm{f}}=\:\frac{{\mathrm{f}}_{0}}{\varDelta\:\mathrm{F}}$$

**Fig. 8:**
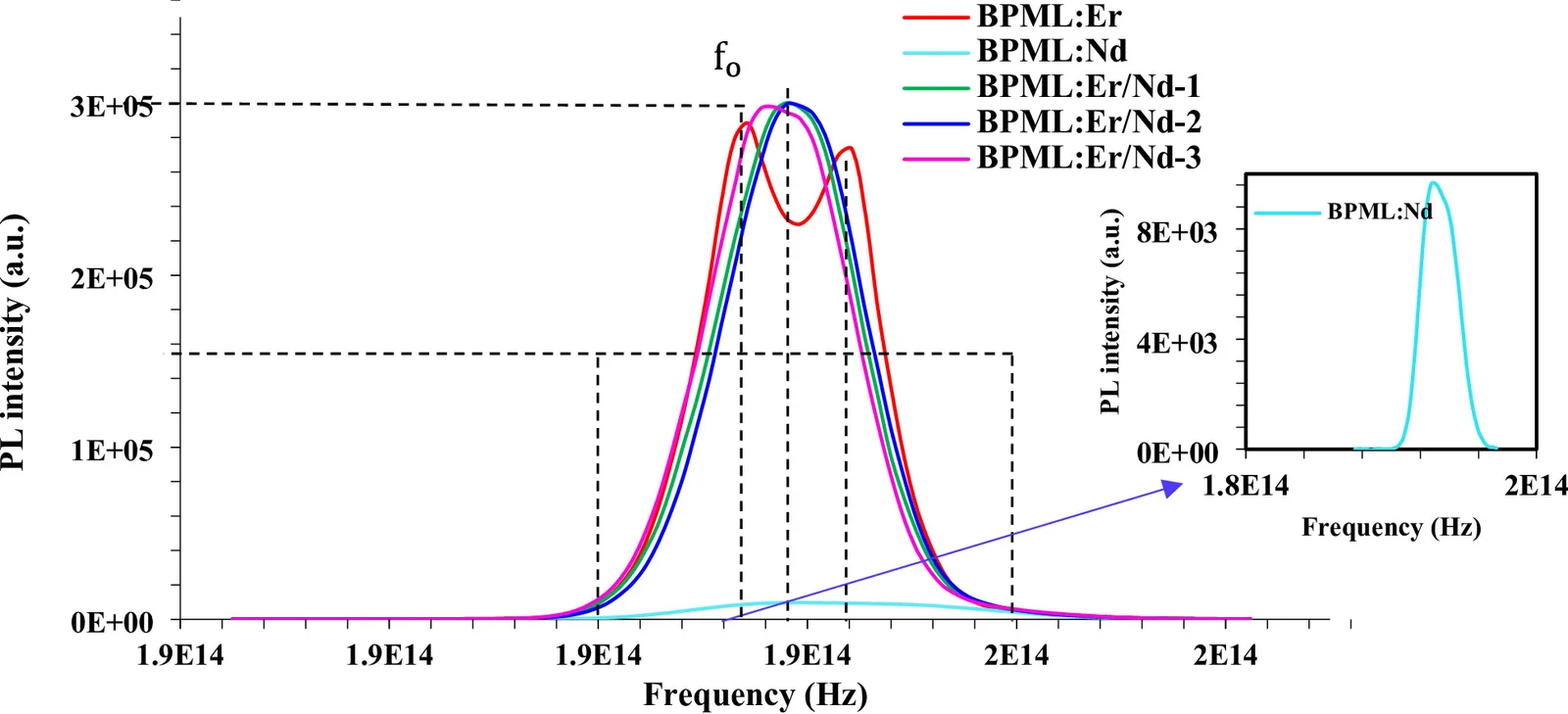
The emitted frequency of the synthesized samples BPML, BPML: Er, BPML: Nd, BPML: Er/Nd-1 BPML: Er/Nd-2, and BPML: Er/Nd-3.

All samples exhibited emission near 1.55 μm, with center frequencies ranging from $\:1.91\times\:{10}^{14}$ to $\:1.96\times\:{10}^{14}$ Hz, as shown in Figure 8, corresponding to the ^4^I_13/2_→^4^I_15/2_ transition of Er^3+^ ions. Although slight variations in the center frequency were observed among the investigated samples (≈ 2.5%), the relatively small magnitude of these changes necessitates a cautions interpretation because instrumental resolution and spectra fitting uncertainty. The observed shifts may therefore reflect subtle modifications in the local glass environment, including variations in structural disorder, phonon interactions, and rare earth distribution within the host matrix. In rare earth doped glasses, Stark splitting effects are typically on the order of 100–300 cm^− 1^, whereas the present frequency variations are considerably larger and my additionally include contributions from spectral broadening, local structural heterogeneity, phonon coupling, and compositional disorder within the glass network. Therefore, no direct quantitative crystal-field assignment is made in the present work. The single-doped BPML: Er and BPML: Nd samples yielded similar quality factors of 38.97 and 38.34, indicating comparable levels of inhomogeneous broadening within the host network. Here, the quality factor ($\:{\mathrm{Q}}_{\mathrm{f}}$) is employed as a spectroscopic parameter that correlates the emission center frequency with the effective spectral bandwidth of the luminescence band. Consequently, higher $\:{\mathrm{Q}}_{\mathrm{f}}$ values are associated with narrower emission bands that are advantageous for broadband again media and wide-band optical amplification applications. In contrast, the Er/Nd co-doped glasses exhibited slightly lower $\:{\mathrm{Q}}_{\mathrm{f}}$ values (≈ 35.2), indicating enhanced emission broadening after Nd^3+^ incorporation. This behavior is attributed to increased structural disorder and additional Nd^3+^→ Er^3+^ energy transfer interactions, which contribute to inhomogeneous broadening of the emission profile. The measured bandwidth ($\:\varDelta\:F\approx\:4.95$ THz) corresponds to approximately 38 nm around 1550 nm, which lies within the technologically important C-band region used in the optical fiber communication systems. Such broadened emission bands are advantageous for wavelength-division multiplexing (WDM) and broadband erbium-doped fiber amplifiers because wider gain bandwidths can support a larger number of optical communication channels and enhance transmission capacity. According to the Shannon-Hartley^78–80^ relationship, increasing the available spectral bandwidth can theoretically increase the channel information capacity, provide that an adequate signal-to-noise ratio is maintained. Therefore, although the co-doped samples exhibit slightly lower spectral purity compared with the single-doped glasses, their broader emission bandwidth may provide practical advantages for broadband photonic and telecommunication applications.

To evaluate the practical applicability of the synthesized BPML: Er/Nd borofluoride glass, its performance was compared with standard silica- and tellurite-based optical fiber materials. High spectroscopic quality factors ($\:{Q}_{f}=35.2-38.97$) and broad emission bandwidths ($\:\varDelta\:F\approx\:4.95$ THz $\:\approx\:38$ nm) spanning the 1.53–1.56 μm region represent key practical advantages over commercial silica fibers ($\:{Q}_{f}=18-20$)^81–83^, significantly benefiting WDM and broadband amplification systems. Furthermore, Nd^3+^ → Er^3+^ energy transfer enhances 1.55 μm emission efficiency under 773 nm excitation, while favorable elastic moduli ($\:E\approx\:45.9-47.4$ GPa) and thermal stability provide processing advantages over conventional tellurite matrices^84–86^. Conversely, practical limitations include lower tensile strength relative to fused silica cores ($\:\sim5$ GPa), requiring polymer cladding during fiber drawing^81^. Additionally, the higher phonon energy of BPML compared to pure tellurite glasses increases non-radiative relaxation rates, necessitating precise concentration control to optimize gain profiles and prevent quenching^84–86^.

Compared with previously reported Er^3+^/Nd^3+^-doped glass systems (Table 8), the present BPML: RE^3+^ glass exhibits a well-balanced combination of thermal, predictive mechanical, and optical properties. The developed borate-rich oxyfluoride composition demonstrates good thermal stability together with enhanced predicted mechanical performance, while maintaining efficient efficient NIR luminescence. In contrast to several reported systems that either require multiple rare earth dopants, employ more compositionally complex glass matrices, or exhibit emission predominantly at shorter NIR wavelengths, the present glass achieves intense emission at 1550–1557 nm under 773 nm excitation using only 1 mol% ER^3+^, Nd^3+^, or their co-doping. The Er^3+^/Nd^3+^ co-doped composition exhibits broadened NIR emission resulting from efficient energy transfer between Nd^3+^ and Er^3+^, together with a high-quality factor (Q_f_ = 38.97). These combined characteristics demonstrate that the BPML: RE^3+^ glass system offers a competitive balance between structural stability and optical performance, making it a promising candidate for broadband optical amplifiers, NIR photonic devices, and providing a solid foundation for future optical fiber applications.

**Table 8:** Comparison of the photoluminescence characteristics of the present BPML: Er/Nd composite with the related Er^3+^/Nd^3+^-doped based luminescent systems.

Material	Thermal properties	Mechanical properties	Excitation (nm)	Emission (nm)	Optical performance	Distinct advantages
60B_2_O_3_-20Pb_3_O_4_-10MgF_2_-10LiF doped with 1 mol% Er^3+^, Nd^3+^, or Er^3+^/Nd^3+^ (Present Work)	Good thermal stability; BPML: Er/Nd-2 exhibits the best thermal balance	$\:\mathrm{E}=45.9-47.5\:\mathrm{G}\mathrm{P}\mathrm{a},\:\mathrm{H}=3.03-3.09\:\mathrm{G}\mathrm{P}\mathrm{a}\:$	773	1550 (Er), 1550 (Nd), 1557 (Er/Nd)	Broadband NIR emission, efficient E^r3+^→Nd^3+^ energy transfer, Q_f_=38.97.	Simple borate-rich oxyfluoride glass with good thermal stability, enhanced predicted mechanical properties, and broadband 1.55 μm emission.
TeO_2_-ZnO-WO_3_-Bi_2_O_3_ doped with Nd^3+^, Tm^3+^, Er^3+23^	$\:\varDelta\:\mathrm{T}=138$°C, excellent thermal stability	ـــــــــ	808	1340, 1480, 1530	Broadband NIR (1300–1630 nm)	Excellent broadband emission but requires triple rare-earth doping and higher compositional complexity.
50B_2_O_3_-20PbO-30CaO-0.5Er_2_O_3_-xNd_2_O_3_ (x = 0.25–1.5 mol% Nd_2_O_3_)^87^	ـــــــــ	ـــــــــ	808	840–1010 (centered at 885)	Nd-related broadband emission	Suitable mainly for radiation-shielding applications rather than telecommunication-band emission
44P_2_O_5_-15ZnO-10Pb_3_O_4_-15NaF-15MgF_2_-1Er_2_O_3_ co-doped with Yb^3+^, Nd^3+^ or Ce^3+88^	$\:{\mathrm{T}}_{\mathrm{g}}=388-403\:^\circ\:\mathrm{C};\:\varDelta\:\mathrm{T}\:=\:362-367\:^\circ\:\mathrm{C}\:(\mathrm{Y}\mathrm{b}/\mathrm{N}\mathrm{d})$ * 188-190*°C (Ce)	Increased elastic moduli and hardness	525	631, 748, 801, 1034, 1527	Red and NIR emission through multi-ion energy transfer	Efficient visible and NIR laser emission, but requires multiple sensitizer ions
(55-x) B_2_O_3_-10SiO_2_-25Gd_2_O_3_-10CaO-xNd_2_O_3_ (x = 0–2.5 mol%)^24^	ـــــــــ	ـــــــــ	808, 885	903, 1059, 1334	Strong Nd^3+^ laser emission	Suitable mainly for laser applications with discrete Nd3 + transitions

## Conclusion

This work demonstrates that the newly engineered borate-rich oxyfluoride glass system, 60B_2_O_3_-20Pb_3_O_4_-10MgF_2_–10LiF (BPML), strategically doped with Er^3+^, Nd^3+^, and their combinations, delivers a compelling suite of structural, thermal, mechanical, and optical characteristics that position it as a strong contender for next-generation broadband optical fiber technologies. Through comprehensive structural analysis, the rare-earth incorporation, most notably Er^3+^, drives significant network densification via enhanced BO_4_ formation, reduced $\:\mathrm{B}-\mathrm{B}$ separation, and strengthened $\:\mathrm{R}\mathrm{E}-\mathrm{O}$ linkages. These structural refinements translate directly into improved mechanical rigidity and elevated thermal stability, with mixed Er^3+^/Nd^3+^ doping achieving an optimal balance between compactness and flexibility. Optically, the glass series exhibits well-defined and broadened absorption features characteristic of Er^3+^ and Nd^3+^, with the co-doped compositions revealing spectrally rich, superimposed transitions indicative of effective simultaneous accommodation of both ions. Most importantly, photoluminescence studies confirm that 773 nm excitation activates an efficient Nd^3+^→Er^3+^ energy transfer pathway, substantially amplifying the 1.55 μm emission band, an essential wavelength for low-loss optical communication. The broadband emission bandwidth (ΔF = 4.95 THz) and high-quality factor ($\:{\mathrm{Q}}_{\mathrm{f}}$ ≈ 38.97) further underscore the suitability of these glasses for high-performance signal amplification and ultrafast photonic applications. Accordingly, the BPML: RE^3+^ glass series unites robust structural integrity, favorable thermal and predicted mechanical behavior, and superior NIR emission performance within a single, compositionally versatile material platform. The reported mechanical properties represent theoretical predictions obtained using the Makishima-Mackenzie model, providing valuable insight into the composition-structure-property relationships of the investigated glasses. As a future research direction, complementary experimental investigations, including Vickers microhardness and ultrasonic measurements, will be carried out to validate the predicted mechanical properties and further establish the reliability of the theoretical estimations. The demonstrated synergy between Er^3+^ and Nd^3+^, manifested in enhanced energy transfer efficiency and broadened emission, marks a significant advancement in the design of rare-earth-activated borate-based oxyfluoride glasses. These results not only validate the efficacy of the BPML system but also establish a promising foundation for the development of next-generation near-infrared optical fibers and broadband amplifiers, capable of meeting the stringent demands of modern high-capacity communication networks.

## Supplementary Information

Below is the link to the electronic supplementary material.

Supplementary Material 1

## Data Availability

Data will be made available on request. The datasets generated during and/or analysed during the current study are available from the corresponding author on reasonable request.
